# Supplementary material for: Molecular engineering of piezoelectricity in collagen-mimicking peptide assemblies
Source: Nat Commun. 2021 May 11;12:2634. doi: 10.1038/s41467-021-22895-6 (PMC8113556; doi:10.1038/s41467-021-22895-6)
Supplement: Supplementary file 1 — Supplementary Information [file 41467_2021_22895_MOESM1_ESM.pdf]

Supplementary Information

Molecular Engineering of Piezoelectricity in Collagen-Mimicking Peptide Assemblies

Santu Bera[1], Sarah Guerin[2], Hui Yuan[3], Joseph O'Donnell[2], Nicholas P. Reynolds[4,5], Oguzhan Maraba[2], Wei Ji[1], Linda J. W. Shimon[6], Pierre-Andre Cazade[2], Syed A. M. Tofail[2], Damien Thompson[2*], Rusen Yang[3*] and Ehud Gazit[1*]

Supplementary section 1: Additional experimental characterization (Supplementary Figs. 1-3, QNM-AFM and Supplementary Table 1):………………………………………………….page 2

Supplementary section 2: Additional DFT predictions (Supplementary Tables 2-7 and Supplementary Fig. 4…………………………………………………………………….page 7

Supplementary section 3: Explanation and justification of PFM results (including Supplementary Figs. 5-16)………………………………………………………………………………page 10

Supplementary section 4: Explanation and justification of MD results (including Supplementary Figs. 17-23 and Supplementary Table 8):………………………………………………….page 21

Supplementary section 5: Additional electrical measurements including Phe-Phe dipeptide control (Supplementary Figs. 24-29 and Supplementary Table 9):……………………………………………………………………………………..page 28

Supplementary section 6: Crystal structure and DFT predicted piezoelectric response of Hyp-Leu-Phe and characterization of tripeptides (including Supplementary Figs. 30-37 and Supplementary Table 10-12):…………………………………………………………Page 34

Supplementary section 1

<u>Additional experimental characterization (Supplementary Figs. 1-3 and Supplementary Table 1):</u>

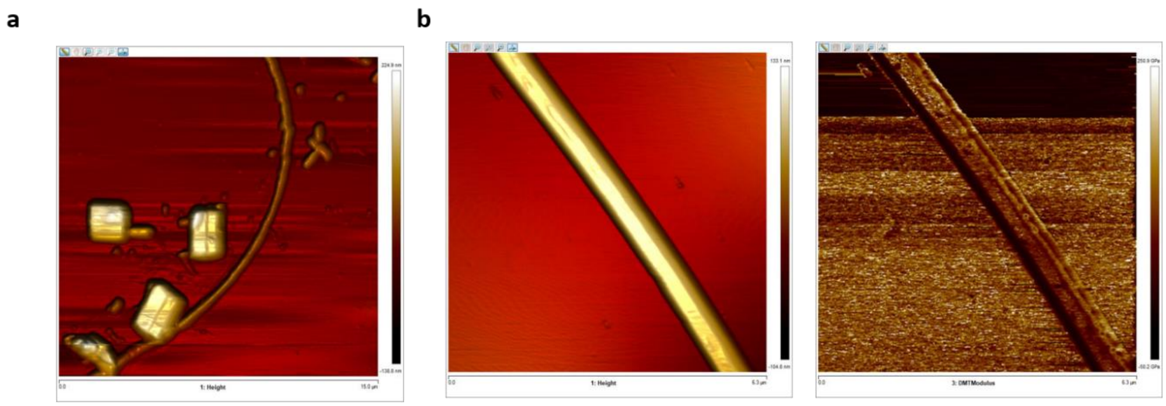

Supplementary Fig. 1| a, AFM images of Hyp-Phe-Phe on silica substrate showing the presence of fibre structure along with some nanocrystals. The vertical width of the image is 15 µm. **b**, Topographic AFM images of Hyp-Phe-Phe fibrils, Z-scale = 133 nm (left side) and Z-scale = 250 GPa (right side).

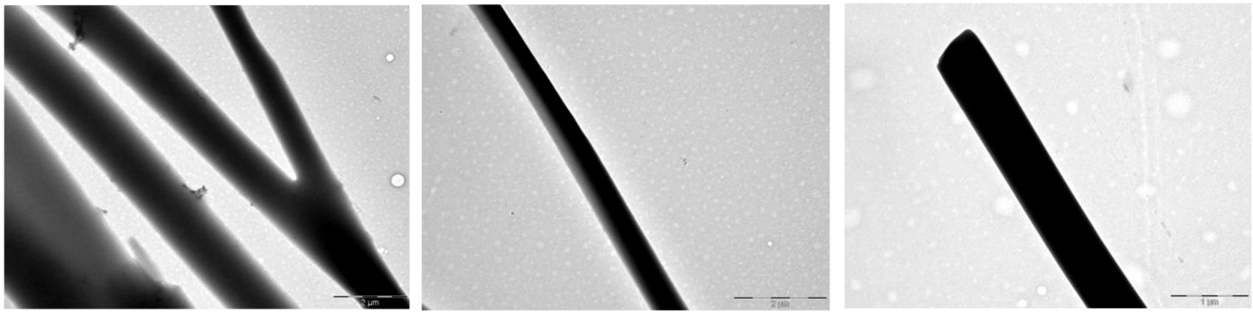

Supplementary Fig. 2| TEM images of Hyp-Phe-Phe fibres.

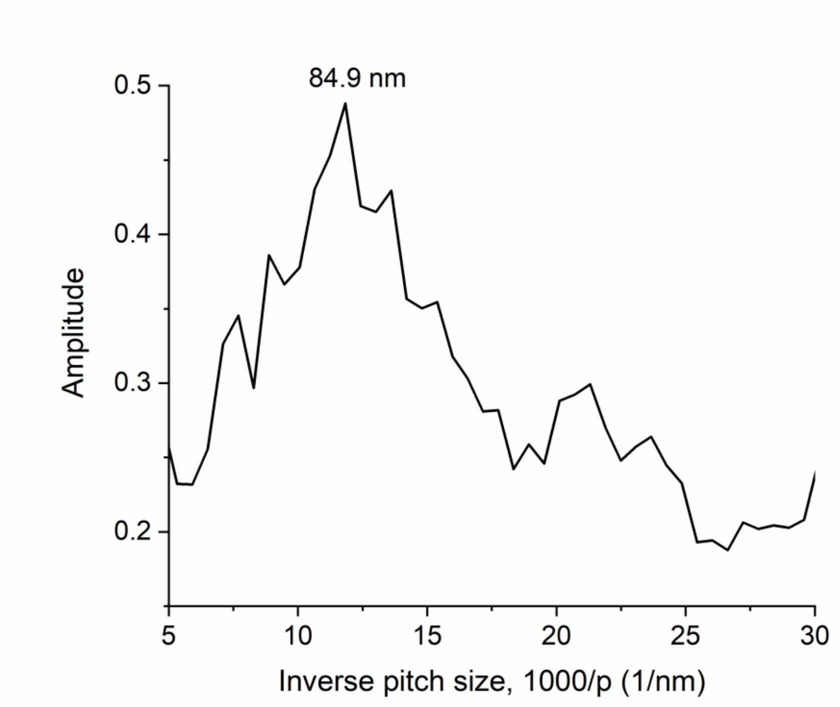

Supplementary Fig. 3| Determination of vertical periodicity in Hyp-Phe-Phe fibres by direct Fourier transform analysis using the open source software FiberApp.

Quantitative Nanomechanical Atomic Force Microscopy (QNM-AFM). In QNM-AFM mode, the cantilever oscillates at a frequency much lower than its resonant frequency. Typically, oscillation frequencies used were 1 or 0.5 kHz and force-separation curves are measured and analyzed for every pixel generated. To ensure accurate quantitative modulus values are recorded, each cantilever is carefully calibrated according to the absolute method as defined by Bruker. Briefly, before imaging, each cantilever was calibrated to accurately determine the deflection sensitivity, spring constant, and the tip radius. Deflection sensitivity was calculated from a force curve generated by indenting the tip into a hard, fused silica surface. The spring constant was determined using the thermal tune procedure included in the Nano Scope control software (V1.4, Bruker). Briefly, a thermal noise spectrum is generated by monitoring small thermal oscillations of the cantilever over a range of frequencies. This thermal noise spectrum can then be used to

numerically calculate the resonant frequency and the spring constant of the cantilever.[1] The tip radius was calculated by analysis of a 1.5 μm scan of a roughened titanium control sample (Bruker, RS-12M) using the tip analysis tool in the NanoScope Analysis software (V1.7, Bruker) and was typically founds to be around 8 nm. While scanning, the deformation channel was carefully monitored to ensure that the maximum indentation into the Hyp-Phe-Phe fibrils did not exceed 2 nm (less than 1 % the thickness of a typical Hyp-Phe-Phe fibril), thus minimizing any substrate influence on the calculated Young's modulus.[2] Nanoscale deviations from planarity in the prepared substrates were subtracted from the topographical maps using the 1st order flatten tool in the Nanoscope Analysis software with no further processing. Nanomechanical maps were generated by analyzing the force-indentation curves using the Derjaguin–Muller–Toporov (DMT) model (equation 1), in order to determine a quantitative value for the reduced Youngs modulus at every pixel.[3]

$$F - F_{adh} = \frac{4}{3}E^{*}\sqrt{R(d-d_0)^3} \text{ ………..(1)}$$

Where $(F - F_{adh})$ is the difference in the force exerted by the AFM tip and the adhesion force, $(d - d_0)$ is the sample deformation, R is the tip radius and E^{*} is the reduced Young's modulus. QNM-AFM uses DMT contact mechanics to routinely calculate the nanomechanical properties of substrates with elastic moduli between 700 kPa and 70 GPa,[3] however it does make a number of assumptions. DMT theory uses modified Hertzian contact mechanics but also accounts for attractive surface forces, however it assumes that these attractive forces act only outside the tip-sample contact area and do not affect deformation. Additionally, DMT theory assumes that the nanoscale morphology of the AFM tip is spherical. Statistical analysis of lateral periodicity

apparent in the AFM data was performed by direct Fourier Transform analysis using the Matlab-based open-source software FibreApp.[4]

Supplementary Table 1. Piezoelectric coefficients of various biological, organic and inorganic materials. We exclude very simple biomaterials made from just one amino acid. The measurement or prediction method used in this work is given in parentheses after the materials together with the index of the individual max d_{ij} coefficient given in parentheses after the value.

Class of material	Material	Piezoelectric coefficient (pm/V)	Supplementary Reference
Biomaterials	Pro-Phe-Phe (DFT prediction)	1.9 (d_{22})	This work
	Hyp-Phe-Phe (DFT prediction)	27.3 (d_{35})	
	Hyp-Leu-Phe (DFT prediction)	3.6 (d_{34})	
	Pro-Phe-Phe (QFN-AFM measurement)	2.2 (d_{33})	
	Hyp-Phe-Phe (QFN-AFM measurement)	4.0 (d_{33}), 16.1 (d_{34})	
	Bone	0.1 (d_{33})	5
	Collagen microfibril	2.64 (d_{33})	6
	Type I collagen	1.1 (d_{15})	7
	Fish skin collagen	5.6 (d_{33})	8
	Collagen triple helix	12 (d_{14})	9
	Collagen peptide	10 (d_{14})	10
	M13 bacteriophage film	11 (d_{33})	11
	Vertically aligned phage nanopillars	6 (d_{33})	12
	α-helical poly(α-amino acid)	25 (d_{33})	13
	Poly-L-lactic acid	15 10 (d_{14})	14
	FF	9.9(d_{33})	15
	FF aligned	18(d_{33})	16
	Silk thin film	56.2(d_{33})	17
Polymers	Bi-axial Poled polyvinylidene-fluoride (PVDF)	32.5(d_{33})	18

**Inorganic Materials**	CdS	14 (d_{15})	19
	ZnO	12.4 (d_{33})	20
	$LiNbO_3$	69 (d_{15})	21
	AlN Thin Films	5.1 (d_{33})	22
	$(Na_{0.5}Bi_{0.5})TiO_3$-$BaTiO_3$-$(K_{0.5}Na_{0.5})NbO_3$	840 pC/N (d_{33})	23
	$(K_{0.88}Na_{0.12})NbO_3$ films	71 pC/N (d_{31})	24
**2D / nano-structured materials**	MnO_2 nanorods/PVDF hybrid films	38 pC/N (d_{33})	25
	PMMA/ZnO NWs	26 pC/N (d_{33})	26
	ZnO Nanorods	9.5(d_{33})	27
	PZT Nanoshells	90(d_{33})	28
	GaN Nanowires	12.8(d_{33})	29
	SnSe	251 (d_{11})	30
	GeS	75 (d_{11})	31

Supplementary section 2:

Additional DFT predictions (Supplementary Tables 2-7 and Supplementary Fig. 4):

Supplementary Table 2: Computed relative permittivity and dielectric constants of Pro-Phe-Phe and Hyp-Phe-Phe single crystals.

Elastic Stiffness Constant	**Pro-Phe-Phe**	**Hyp-Phe-Phe**
c_{11}	22.4	22.9
c_{22}	21.6	26.1
c_{33}	22.6	21.9
c_{44}	2.0	1.7
c_{55}	8.7	3.6
c_{66}	3.8	1.1
Young's Modulus	14	12

Supplementary Table 3: Computed elastic constants of Pro-Phe-Phe and Hyp-Phe-Phe single crystals. All values are in GPa.

Dielectric Constant	**Pro-Phe-Phe**	**Hyp-Phe-Phe**
ε_1	3.03	2.89
ε_2	3.04	3.05
ε_3	3.18	3.97
ε_r	3.08	3.30

Supplementary Table 4: Calculated piezoelectric charge tensor components e_{ij} (in units of C/m^2), strain tensor components d_{ik} (pm/V), and voltage tensor components g_{ij} (mV m/N), of Pro-Phe-Phe.

Charge Tensor (C/m^2)

$$\begin{pmatrix} 0 & 0 & 0 & 0.005 & 0 & -0.005 \\ -0.056 & 0.041 & -0.018 & 0 & -0.009 & 0 \\ 0 & 0 & 0 & 0.014 & 0 & -0.009 \end{pmatrix}$$

Strain Tensor (pm/V)

$$\begin{pmatrix} 0 & 0 & 0 & 2.4 & 0 & -1.4 \\ -2.5 & 1.9 & -0.8 & 0 & -1.0 & 0 \\ 0 & 0 & 0 & 3.1 & 0 & -2.4 \end{pmatrix}$$

Voltage Tensor (mV m/N)

$$\begin{pmatrix} 0 & 0 & 0 & 81 & 0 & -48 \\ -86 & 65 & -27 & 0 & -34 & 0 \\ 0 & 0 & 0 & 108 & 0 & -83 \end{pmatrix}$$

Supplementary Table 5: Calculated piezoelectric charge tensor components e_{ij} (in units of C/m^2), strain tensor components d_{ik} (pm/V), and voltage tensor components g_{ij} (mV m/N), of Hyp-Phe-Phe.

Charge Tensor (C/m^2)

$$\begin{pmatrix} 0 & 0.003 & -0.019 & 0.006 & 0.039 & -0.08 \\ 0.048 & -0.026 & 0.004 & 0.007 & 0 & 0 \\ -0.053 & 0.055 & -0.102 & -0.016 & 0.108 & -0.004 \end{pmatrix}$$

Strain Tensor (pm/V)

$$\begin{pmatrix} 0 & 0.2 & -0.1 & 3.5 & 0.6 & 27.3 \\ 2.4 & -1.3 & -0.1 & -2.2 & -1.4 & 0.7 \\ -0.1 & 0.3 & 4.82 & -2.2 & -27.3 & 17.1 \end{pmatrix}$$

Voltage Tensor (mV m/N)

$$\begin{pmatrix} 0 & 7 & -5 & 127 & 21 & 1043 \\ 86 & -3.9 & -3 & -77 & -49 & 23 \\ -4 & 12 & 130 & -74 & -996 & 474 \end{pmatrix}$$

Supplementary Table 6. Predicted crystal dipole moments obtained as described in main text Methods.

	X	Y	Z
Pro-Phe-Phe	0.000	-2.762	0.000
Hyp-Phe-Phe	0.142	-1.918	-0.219

Supplementary Table 7. Predicted binding energies between molecules in the hydroxylated and non-hydroxylated tripeptide assemblies, calculated as described in main text Methods.

Energy (eV)	Hyp-Phe-Phe	Pro-Phe-Phe
**Pair of molecules (E_p)**	-734.31	-721.58
**Single molecule (E_s)**	-366.16	-359.62
**Binding Energy = E_p – $2E_s$**	-1.99	-2.34

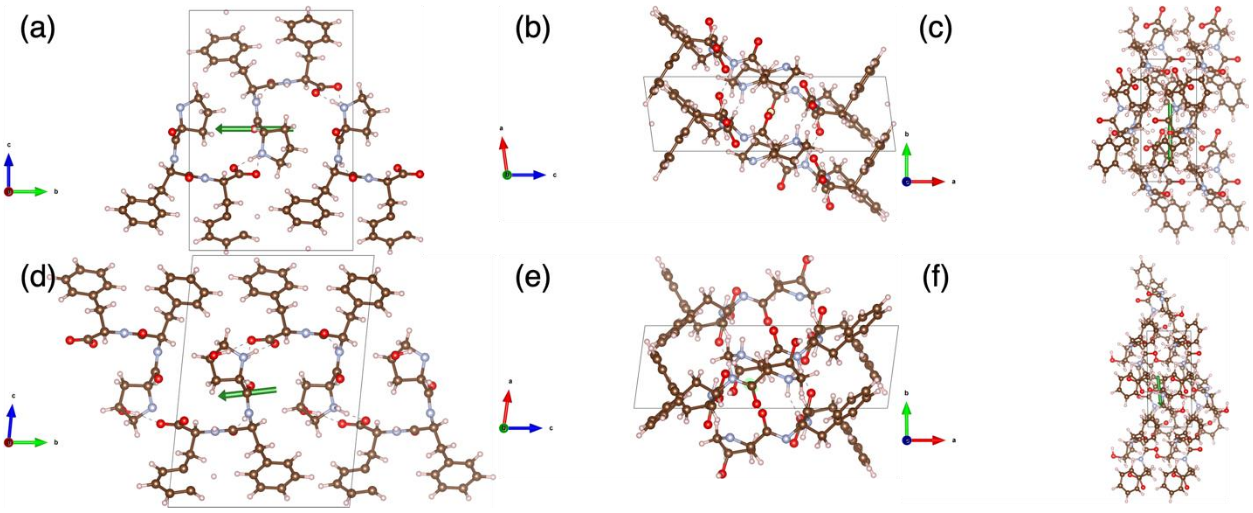

Supplementary Fig. 4| The single crystal unit cells computed using DFT with the CP2K code (see main text Methods) are shown with the crystal dipole moment overlaid as a dark green arrow. Tripeptides are represented by ball and sticks. **a**, **b**, and **c**, depict Pro-Phe-Phe crystal projected along a-axis, b-axis, and c-axis, respectively. **d**, **e**, and **f**, depict Hyp-Phe-Phe crystal projected along a-axis, b-axis, and c-axis, respectively. The dipole moment of both crystals was magnified x2 to make it more visible.

Supplementary section 3:

<u>Explanation and Justification of PFM results (including Supplementary Figs. 5-16):</u>

The operation of PFM is detailed in Supplementary Fig. 5. For these experiments, three single crystals from each sample were selected, and a further three sites along each crystal were then chosen. At each of these three sites, five measurements were taken in a 2 μm^2 area. This resulted in 45 measurements for each material.

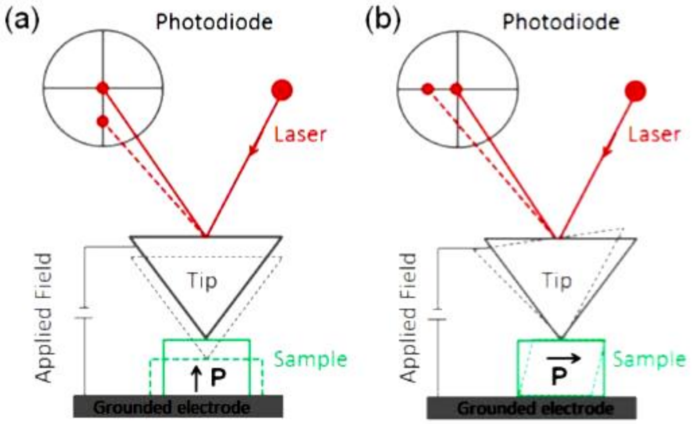

Supplementary Fig. 5| Schematic of PFM operation. A sample deforms in response to an applied AC voltage, which causes the cantilever to deflect. **a**, When the polarisation is parallel to the applied voltage, the sample will generate an out-of-plane piezoresponse, measured by the tip-photodiode system as a vertical deflection. **b**, When the polarisation is perpendicular to the applied voltage, the sample will generate an in-plane piezoresponse that is measured as a lateral deflection by the tip-photodiode system.

Our PFM system allows us to sweep the voltage between 0 V and 50 V and back again and simultaneously measure the magnitude of the vertical piezoresponse detected by the photodiode system. Supplementary Fig.6 shows a representative plot for poled PVDF, which has $d_{33} = 20.7\ pm/V$ according to the manufacturer. This is used as our positive control.

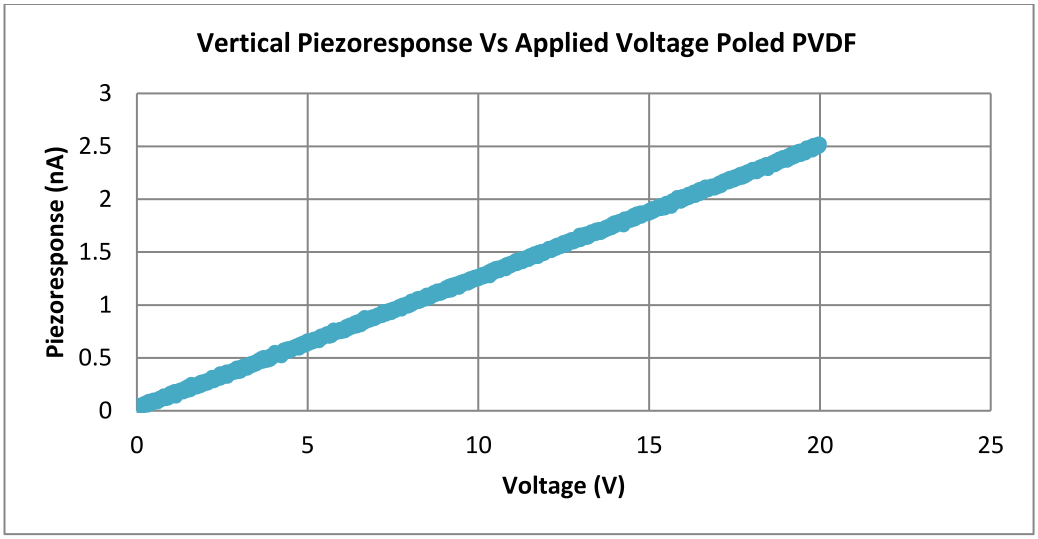

Supplementary Fig. 6| Linear relationship between the vertical piezoresponse as measured by the photodiode system and the applied voltagefor poled PVDF.

This linear relationship indicates a genuine piezoelectric response. It suggests that piezoelectricity is the main contributing factor. The slope of this line is 0.1232 nA/V. This slope is proportional to the d_{33}^{eff} piezoelectric coefficient of the sample. The equation linking these is:

$$d_{33}^{eff}\left(\frac{pm}{V}\right) = \frac{Slope\ of\ piezoresponse\ curve\left(\frac{nA}{V}\right)}{Gain \times Input \times IOS_V\left(\frac{nA}{nm}\right)} \times 1000 \ldots\ldots(2)$$

The IOS_V (vertical inverse optical sensitivity) coefficient is essentially a conversion factor, relating the unit of deformation recorded by the photodiode to the unit needed for quantification. It depends on the alignment of the laser on the tip and the reflectivity of the back surface of the tip. The IOS_V is calculated from the slope of a force-distance curve performed on a hard substrate. Supplementary Fig. 7 shows a force-distance curve taken on a hard substrate. In this case, the IOS_V is 0.061 nA/nm. The Gain and Input are both experimental factors we select when scanning and in this caseboth were equal to 10. Therefore, evaluation of the d_{33}^{eff} piezoelectric

coefficient of poled PVDF yields $d_{33}^{eff} = 20.2\, pm/V$ which is in excellent agreement with the manufacturer's specification. This technique was used to extract all of the d_{33}^{eff} piezoelectric coefficients measured for the peptide crystals. An effective shear piezoelectric coefficient, d_{34}^{eff} can be calculated from the slope of a plot similar to that shown inSupplementary Fig. 7 but with lateral piezoresponse on the vertical axis. These curves can be captured at the same time as the vertical piezoresponse curves. The formula for quantification is the same as above but the IOS_V is replaced with the IOS_L coefficient, which can be derived based on a simple geometrical relationship between the length of the cantilever, L, the height of the tip, h, and the ratio of the vertical sensitivity to the lateral sensitivity, R.

$$R = \frac{IOS_V}{IOS_L} = \frac{2L}{3h} \dots\dots\dots (3)$$

The sensitivity of the lateral response is generally greater (meaning the lateral inverse optical sensitivity is generally smaller) than that of the vertical response. Calibrating the vertical sensitivity as described above, allows the lateral resolution to be estimated. For example, taking an IOS_V of 0.061 nA/nm and applying the equation above assuming a tip height of 15 µm and a cantilever length of 125 µm (these quantities are readily available on the probe packaging) yields an IOS_L of 0.01098 nA/nm. This is almost six times smaller than the vertical inverse optical sensitivity, as expected.

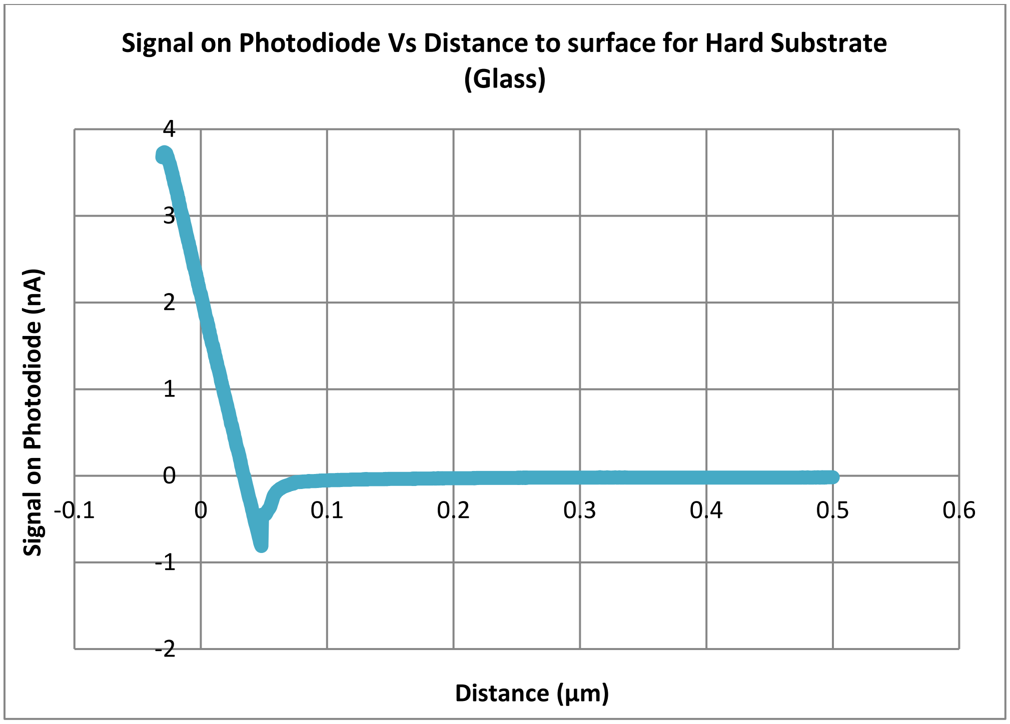

Supplementary Fig. 7| Force-distance curve measuredon a hard glass substrate. The IOS is the slope of the linear part of this curve.

To ensure electrostatic effects coming from both the substrate and the system were not playing any role in the measurements, a number of voltage sweeps were taken on negative control samples, namely copper (Supplementary Fig.8), Kapton (Supplementary Fig.9) and glass (Supplementary Fig.10). This was to ensure both hard, soft, insulating and conductive non-piezoelectric samples were tested for a response using the same setup and parameters as those used for measurements on the peptide crystals. A noisy, nonlinear relationship can be seen in all cases, with the magnitude of the noise being far less than the piezoresponse magnitude on real piezoelectric samples. All voltage sweeps are from 0 V to 20 V. A very small slope can be seen in some cases, indicating that a very small background may be present. This background is of the order of 0.1 pm/V when the appropriate precautions are taken.

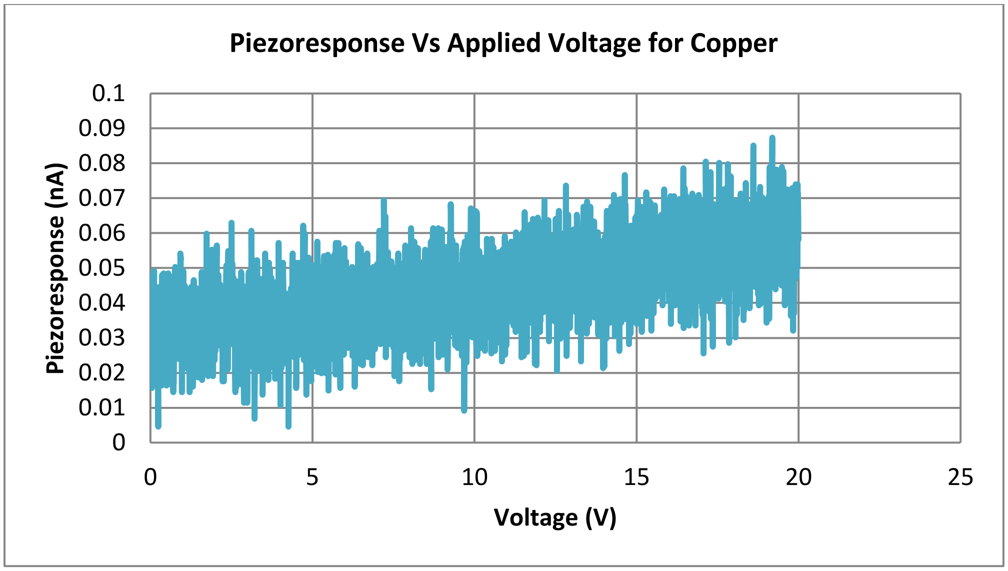

Supplementary Fig. 8| Nonlinear, noisy relationship between the piezoresponse as measured by the photodiode system and the applied voltage for copper substrate.

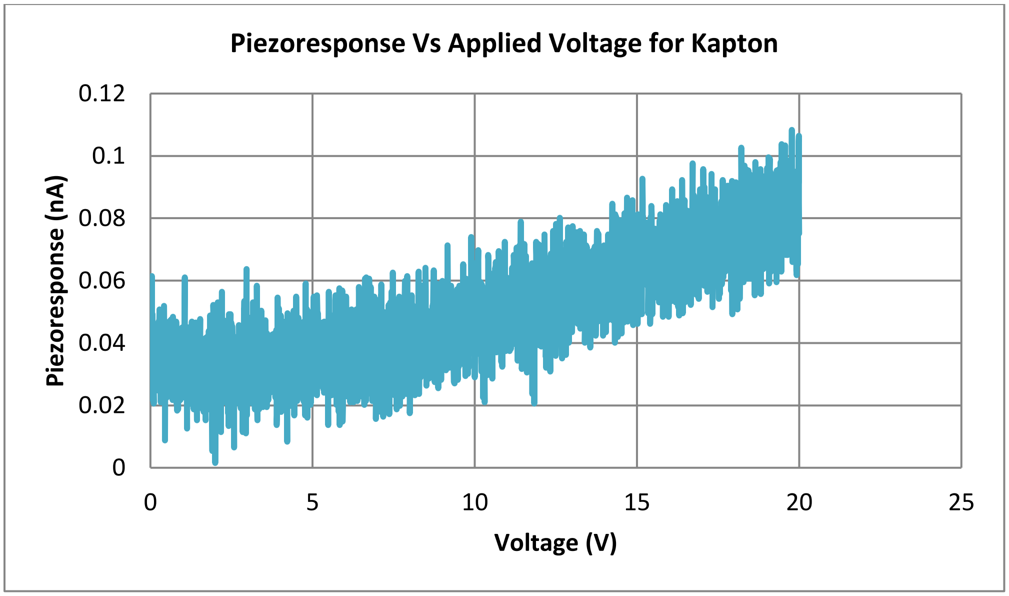

Supplementary Fig. 9| Nonlinear, noisy relationship between the piezoresponse as measured by the photodiode system and the applied voltage for Kapton substrate.

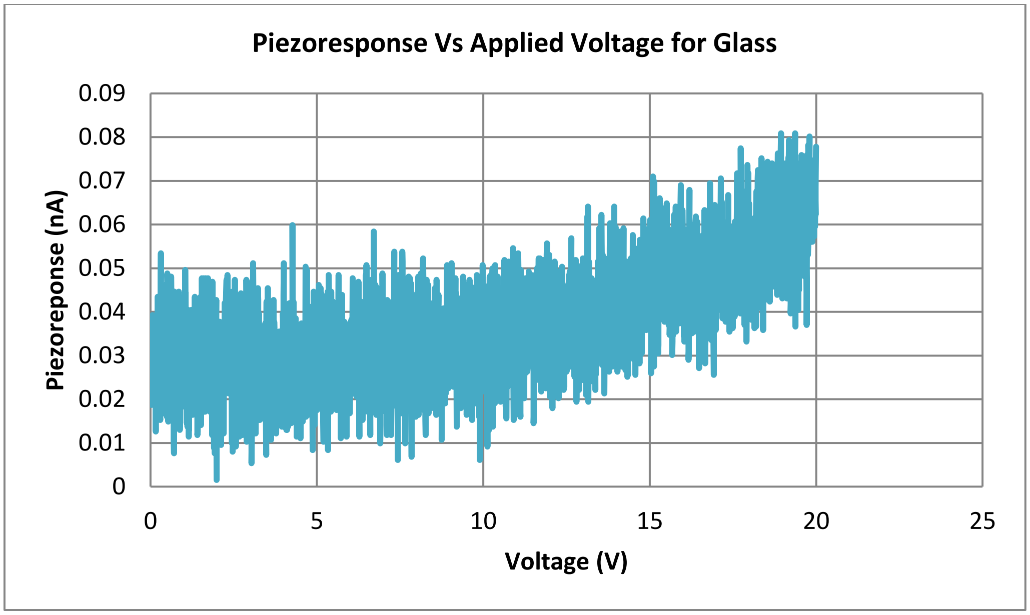

Supplementary Fig. 10| Nonlinear, noisy relationship between the piezoresponse as measured by the photodiode system and the applied voltage for glass substrate.

Once benchmarked using these control experiments, we use the PFM setup to measure and quantitatively compare the piezo responses of the Pro-Phe-Phe and Hyp-Pro-Pro crystals (main text and Supplementary Figs. 11-16 below).

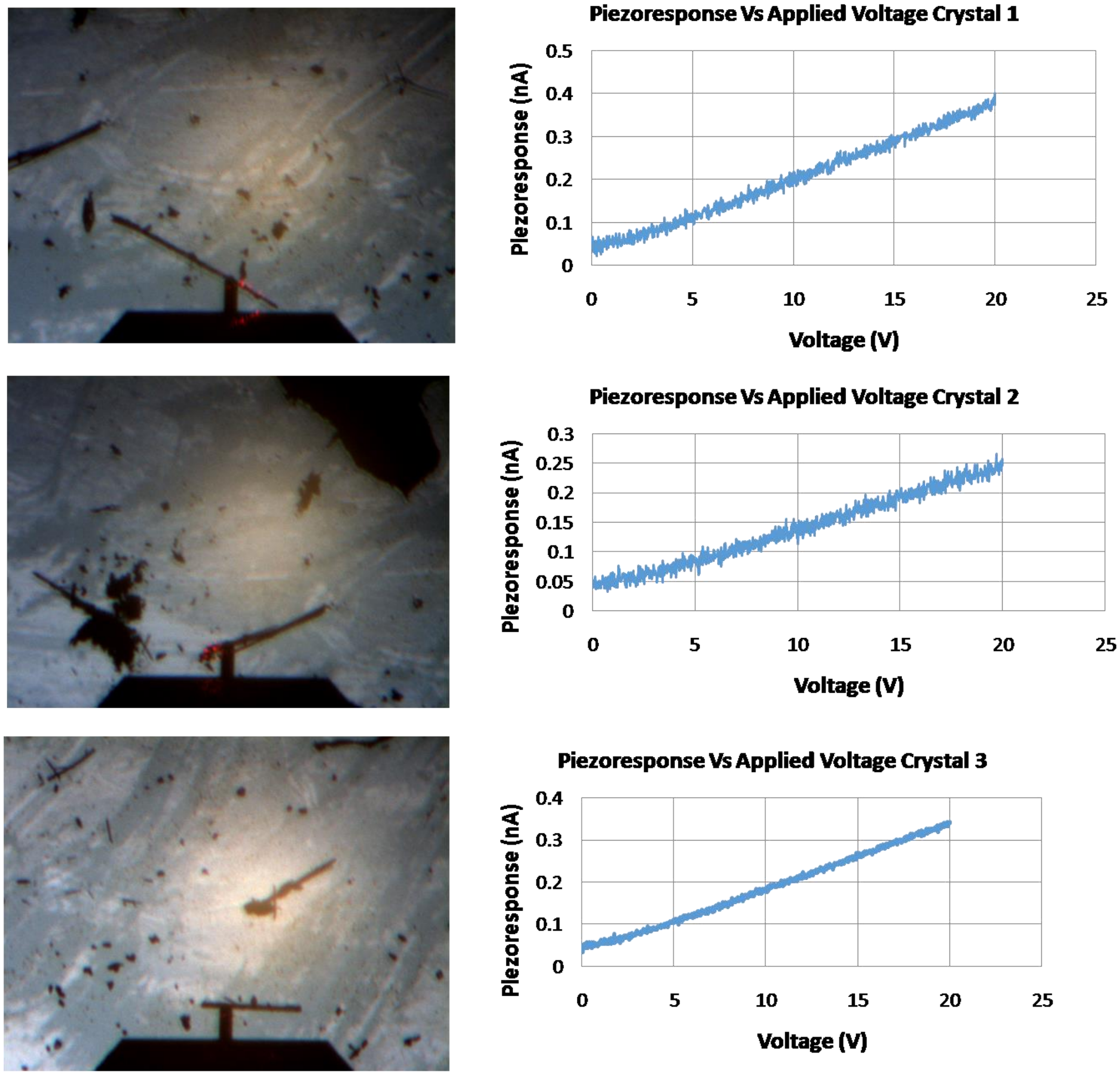

Supplementary Fig. 11| The examined crystals of Pro-Phe-Phe under the PFM tip and the corresponding linear relationship between the vertical piezoresponse as measured by the photodiode system and the applied voltage.

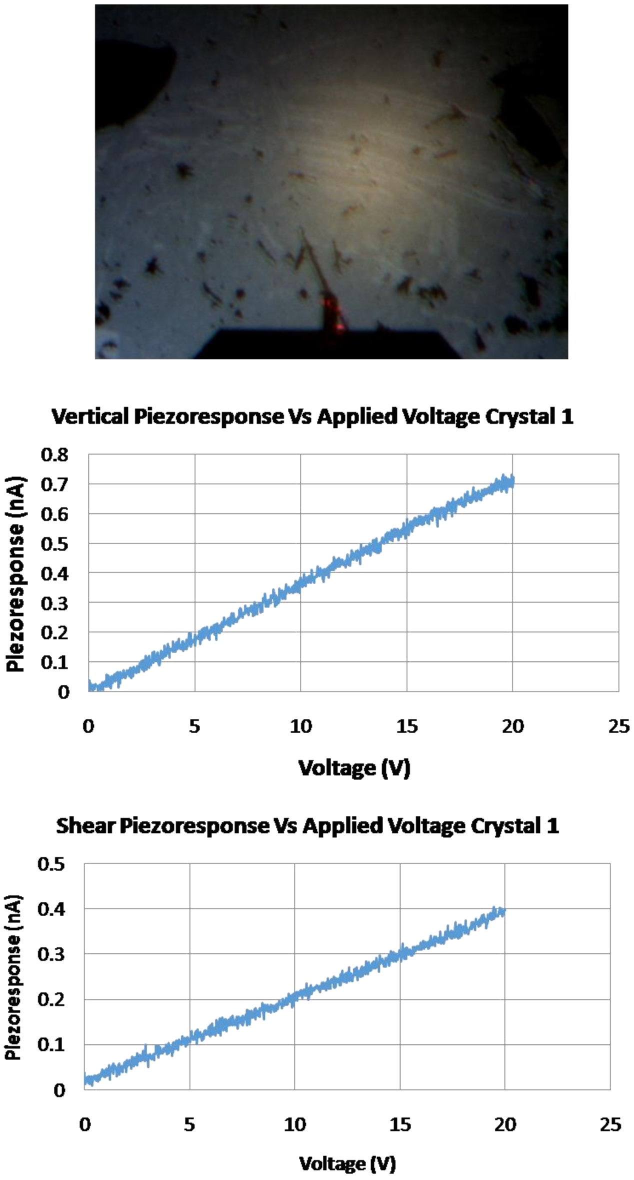

Supplementary Fig. 12| The examined crystals of Hyp-Phe-Phe under the PFM tip. The corresponding linear relationship between the vertical and shear piezoresponse as measured by the photodiode system and the applied voltage.

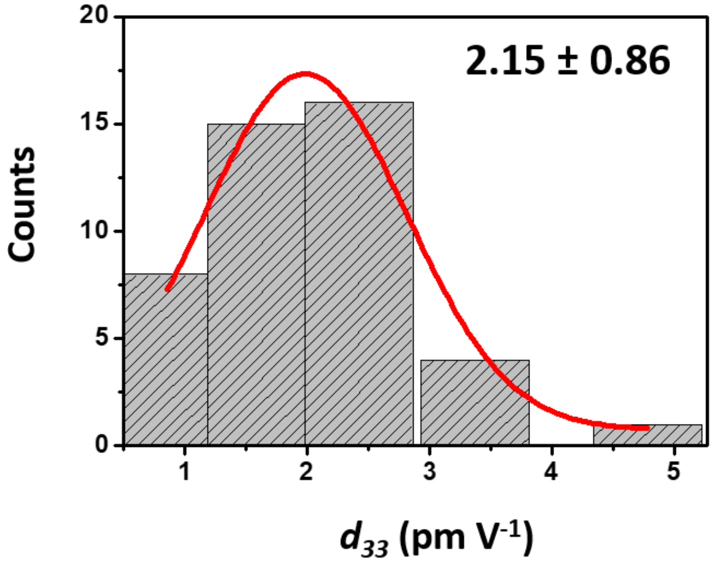

Supplementary Fig. 13| Statistical distribution of the vertical d_{33} coefficients of Pro-Phe-Phe.

Supplementary Fig. 14-16 show the measured frequency dependence of the piezoelectric responses for both the Pro-Phe-Phe and Hyp-Phe-Phe tripeptide crystals. Supplementary Fig. 14 depicts the frequency dependence of the vertical piezoresponse for Pro-Phe-Phe crystals while Supplementary Fig. 15 and 16 respectively show the frequency dependence of the vertical and lateral signals for Hyp-Phe-Phe. All frequency spectra were acquired using a stiff, diamond coated probe with a spring constant of 40 N/m at 20 V applied voltage. Minimal frequency dependence is observed with no significant variations in the smooth piezoresponse present in the region of 20 kHz. This is demonstrative of a genuine piezoelectric response, converged with respect to the frequency of the applied voltage.

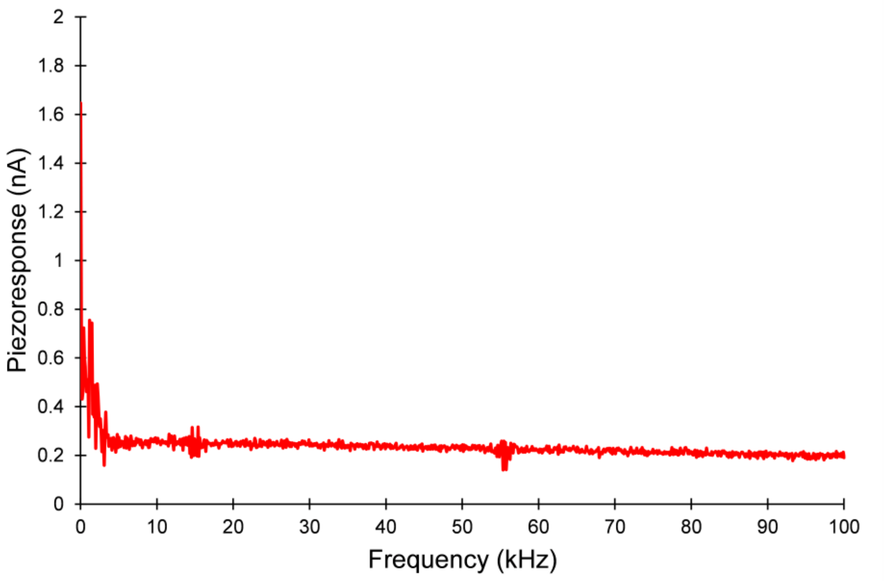

Supplementary Fig. 14: Measured frequency dependence of the vertical piezoresponse for Pro-Phe-Phe.

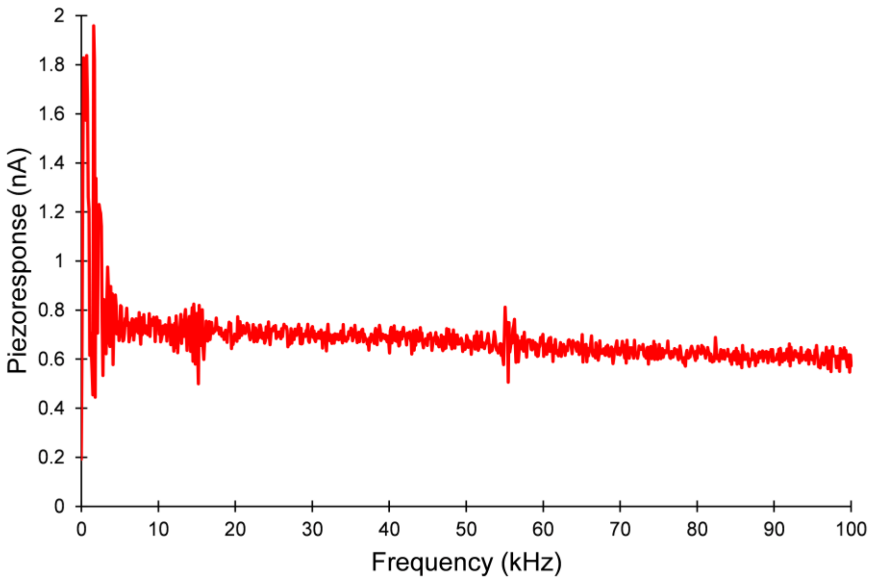

Supplementary Fig. 15: Measured frequency dependence of the vertical piezoresponse for Hyp-Phe-Phe.

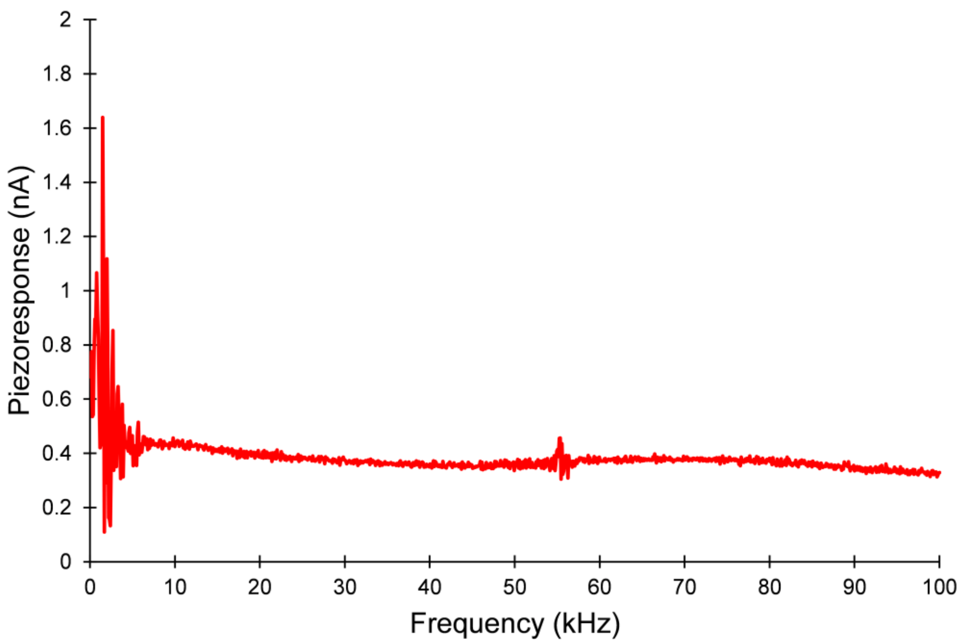

Supplementary Fig. 16: Measured frequency dependence of the lateral piezoresponse for Hyp-Phe-Phe.

Supplementary Section 4.

Explanation and Justification of MD results (including Supplementary Figs. 17-23 and Supplementary Table 8):

The statistical analysis of the ring stacking and H-bond populations computed using molecular dynamics computer simulations is summarised in main text Fig. 3. In the H-bond histograms in main text Fig. 3d the reference XRD measurements are shown as solid vertical lines. The computed distribution of (N-H --- O=C) hydrogen bonds between the C-terminal F residue and two N-terminal P residues of Pro-Phe-Phe are labelled H1 and H2, as shown in Supplementary Fig. 17. The MD values are molecule- and time-averaged distances sampled over 10000 statistically independent structures during 0.1 μs of dynamics for each peptide crystal. The computed distribution of (N-H --- O=C) hydrogen bonds between the C-terminal F residue and its neighbouring 1st F residue is labelled H3. The computed distribution of (N-H --- O=C) hydrogen bonds between the 1st F residue and the neighbouring 1st F residue is H4. H5 is the new (C-OH --- O=C) H-bond due to hydroxylation of Pro to make the Hyp-Phe-Phe peptide. Computed distributions of center of mass distances of F rings are labelled RS for ring stacking.

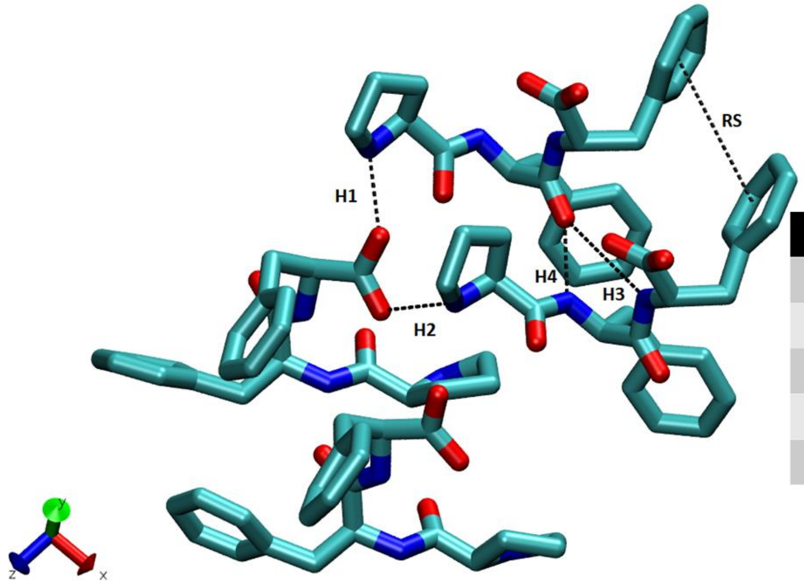

Contact	MD (Å)	XRD (Å)
H1	2.81 ± 0.06	2.98
H2	2.70 ± 0.04	2.87
H3	4.04 ± 0.11	3.54
H4	2.77 ± 0.05	2.77
RS	5.54 ± 0.35	5.32

(a) Pro-Phe-Phe

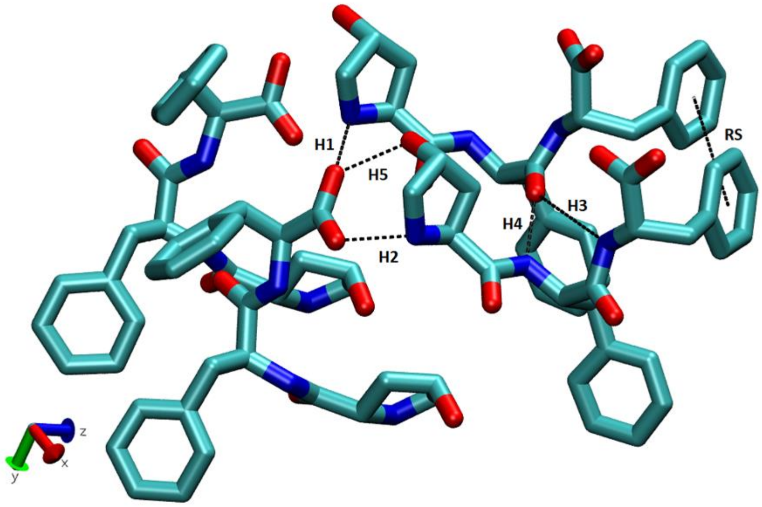

Contact	MD (Å)	XRD (Å)
H1	2.64 ± 0.04	2.75
H2	2.58 ± 0.03	2.65
H3	3.90 ± 0.11	3.73
H4	2.78 ± 0.05	2.78
H5	2.68 ± 0.07	2.77
RS	5.62 ± 0.62	5.43

(b) Hyp-Phe-Phe

Supplementary Fig. 17| Illustration of the labelling used to monitor pairwise H-bonds and ring stacking (RS) interactions in peptide crystals (**a**) Pro-Phe-Phe and (**b**) Hyp-Phe-Phe. Inset tables show the calculated and starting XRD values. Histograms for H-bonds H1-H5 are in main text Fig. 3d and RS π–π distributions are shown in Fig. 3c and Supplementary Figs. 18 and 19 below.

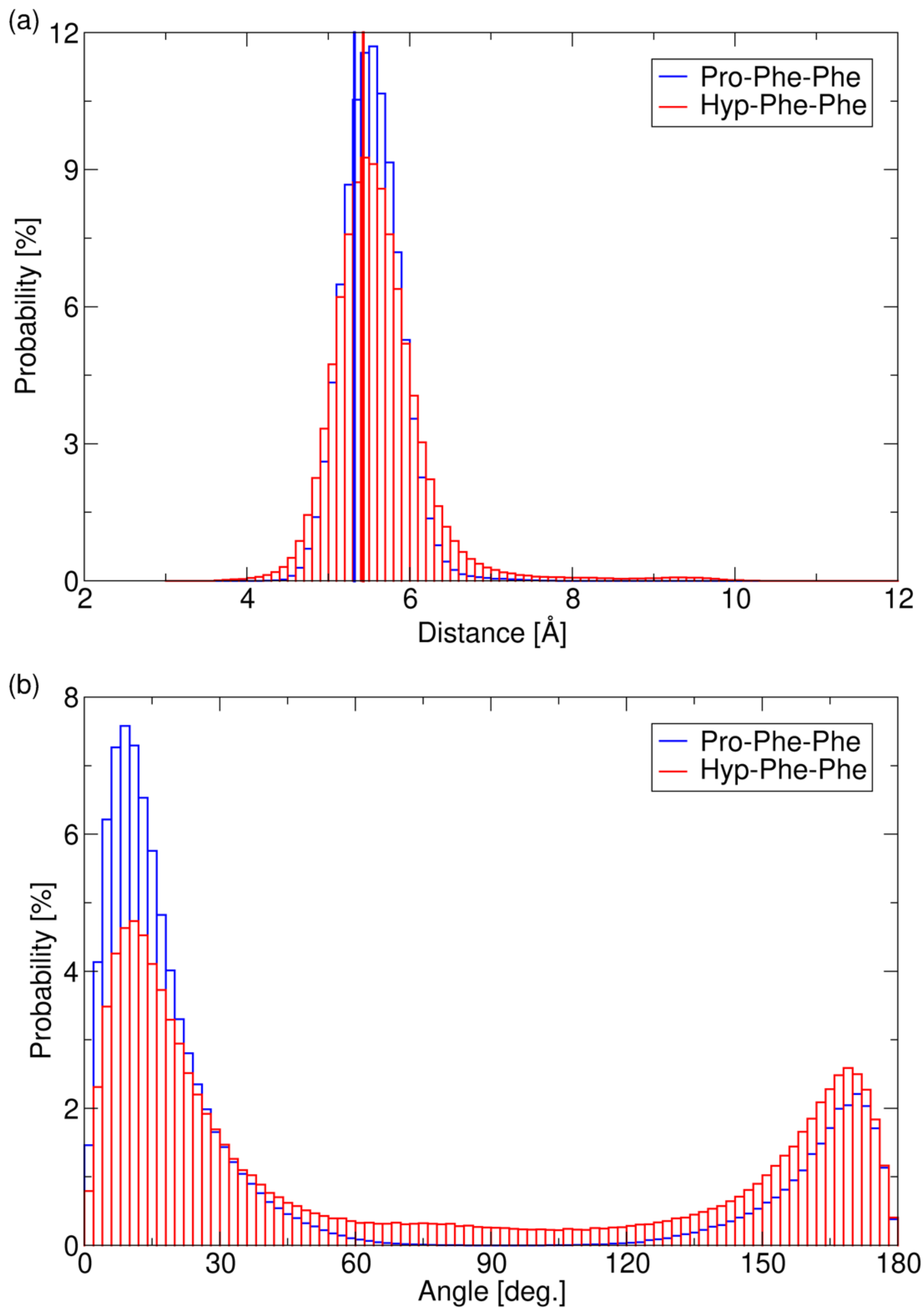

Supplementary Fig. 18| a, Computed distributions of center of mass distances of stacked Phe rings. The blue and red vertical lines indicate the crystal reference value for Pro-Phe-Phe and Hyp-Phe-Phe, respectively. **b**, Computed angles sampled between stacked Phe rings.

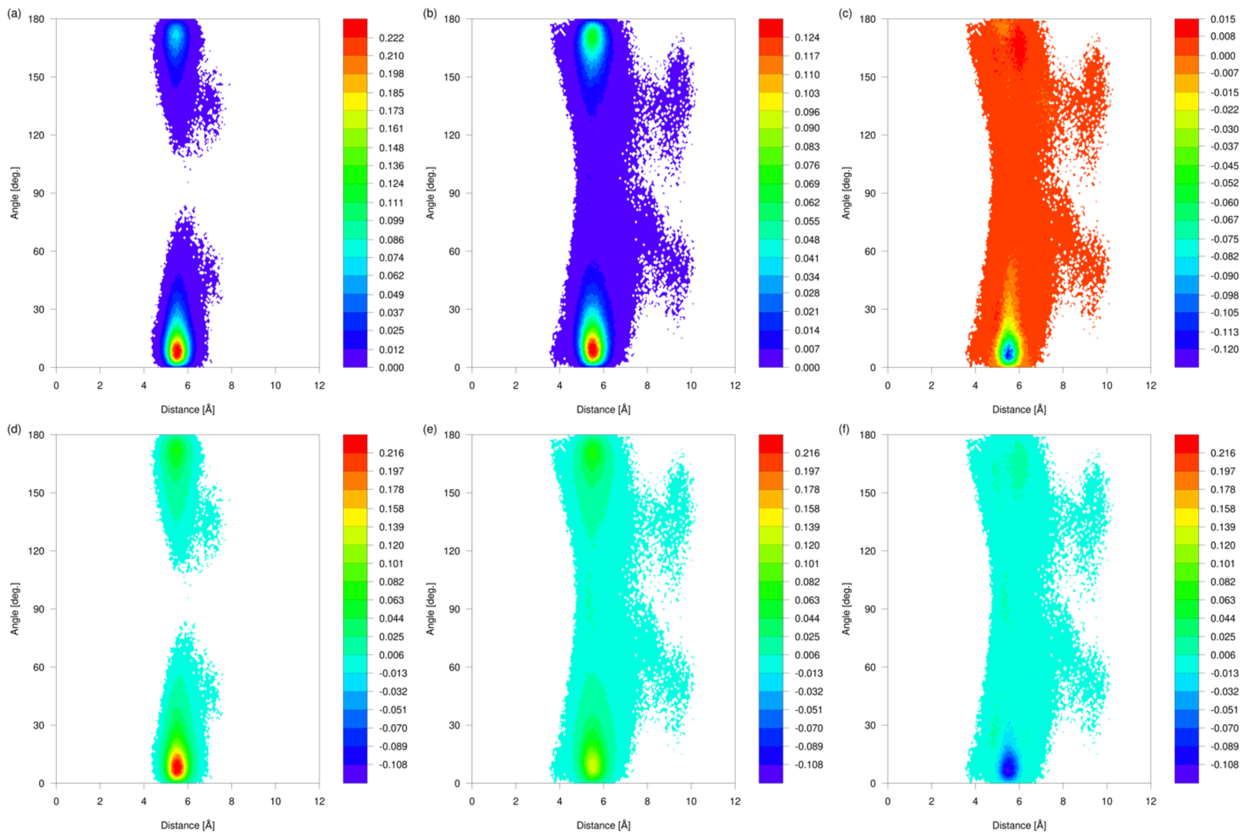

Supplementary Fig. 19| The angle vs. distance density plots for ring-ring contacts in the peptide cystals. **a**,**d**, depicts density for Pro-Phe-Phe. **b**,**e** depicts density for Hyp-Phe-Phe. **c**,**f**, depicts difference between Hyp-Phe-Phe density and Pro-Phe-Phe density. **a**,**b**, and **c**, depicts density with a scale adapted to each plot range to improve contrast. **d**, **e**, and **f**, depicts density on the same scale across the three plots to allow direct comparison.

Supplementary Table 8. Comparison between the experimental XRD lattice parameters and the optimised parameters obtained using the CP2K code with DFT and with the classical CHARMM 36m forcefield with methodologies described in main text Methods.

		A	*B*	*C*	α	β	γ
Pro-Phe-Phe	Exp.	5.321	11.569	17.040	90.00	97.22	90.00
	DFT	5.298	11.534	17.036	90.00	97.72	90.00
	C36m	5.297	11.591	17.214	90.00	96.07	90.00
Hyp-Phe-Phe	Exp.	5.432	11.891	16.765	84.57	83.00	89.87
	DFT	5.457	11.886	16.675	84.39	82.33	89.95
	C36m	5.431	11.845	16.786	87.59	82.09	89.75

Supplementary Fig. 20 shows the final computed structure of the stable, persistent, and tightly-knit zipper motif in (a) Pro-Phe-Phe and (b) Hyp-Phe-Phe following 200 ns of molecular dynamics.

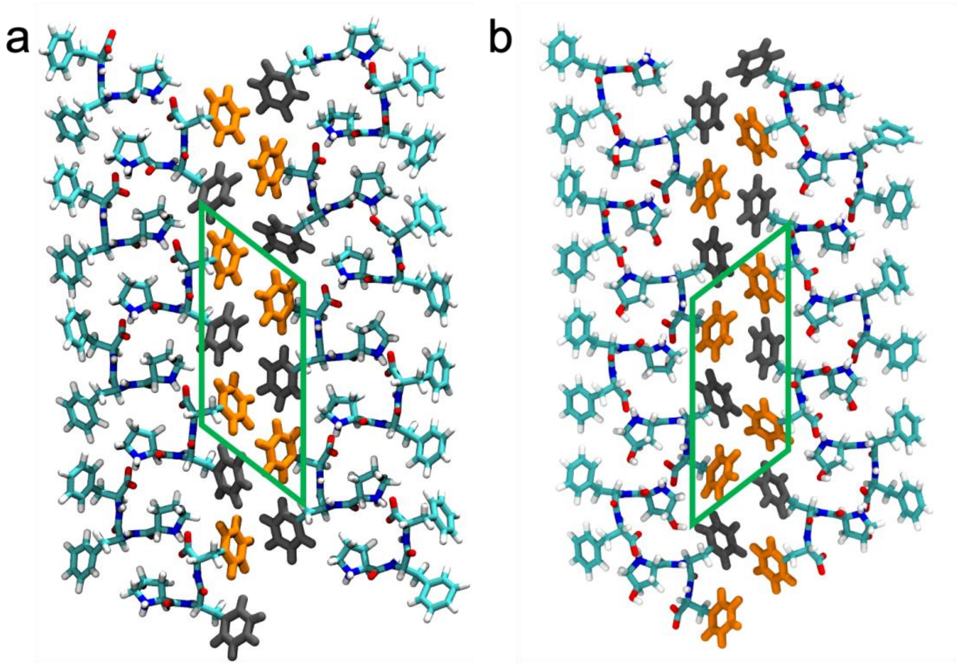

Supplementary Fig. 20| Stick representation of the zipper motif that stabilizes the tripeptide assemblies. The phenyl rings involved in the zipper are depicted in gray for the second residue and orange for the third residue of the tripeptide in (a) Pro-Phe-Phe and (b) Hyp-Phe-Phe. The green shapes indicate the contacts that are selected in the monitoring of zipper stabilization in Supplementary Fig. 21.

The stability of the zippers is confirmed by the net favorable computed interaction energies shown in Supplementary Fig. 21. The extended pi-pi zipper seam creates a strong and stable intra-layer structure, which couples with the stable interlayer π-π stacking (Fig. 3) to create the robust, mechanically-stable tripeptide assemblies.

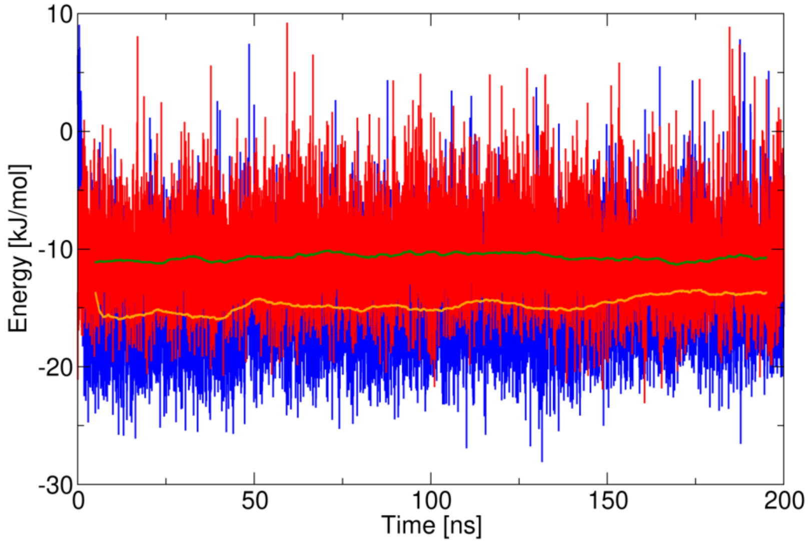

Supplementary Fig. 21| Interaction energies computed over 200 ns within the zipper of Pro-Phe-Phe (blue) and Hyp-Phe-Phe (red), using the 6-Phe sampling units marked in Figure Supplementary Fig. 20. The orange and green curves are the running averages for Pro-Phe-Phe and Hyp-Phe-Phe, respectively.

Supplementary Fig. 22 shows the corresponding distribution of the distance between pairs of phenyl rings across the zipper motif and their relative orientation during the final 50 ns of dynamics. For both tripeptide assemblies the distance distributions are narrow and centered close to the contact distance in the X-ray crystal structures, which together with the ordered orientations confirms the strength and stability of the zipper. Supplementary Fig. 22c,d shows density maps of the distance-angle landscape, illustrating the predominant major population of ordered pi-stacked contacts in the zippers.

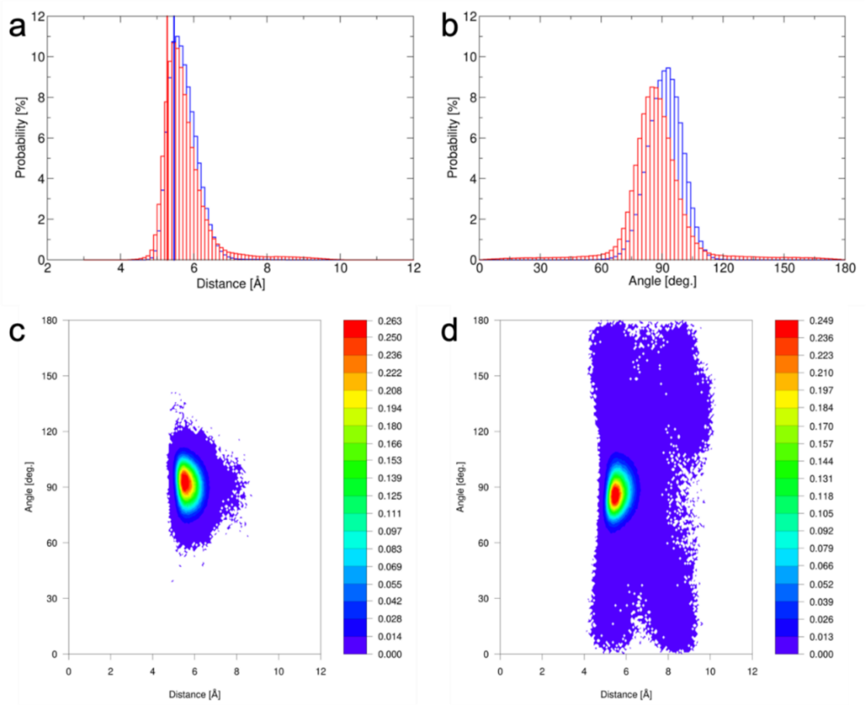

Supplementary Fig. 22| Distributions during the final 50 ns of dynamics of the distance (a) and angle (b) between pairs of phenyl rings that make the zipper. Data for Pro-Phe-Phe is plotted in blue and data for Hyp-Phe-Phe is plotted in red. The distance-angle distributions are characterized in more detail in the density maps in panels (c) Pro-Phe-Phe and (d) Hyp-Phe-Phe.

The measured unit cell parameters of a single crystal with respect to the crystal morphology also helps confirm the specific organization of helices that stabilize the crystal (Supplementary Fig. 23)."

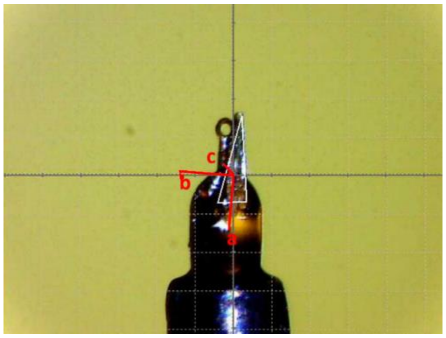

Supplementary Fig. 23| Unit cell measurement of the crystal with respect to crystal morphology. Single crystal is shown mounted on a MiTeGen loop. The crystal is highlighted in white box and the respective cell axes are shown in red. The morphological long axis of the crystal is aligned along the crystallographic *a* axis of the unit cell. Size of the crystal is 0.262 x 0.090 x 0.016 mm.

Supplementary Section 5.

Additional electrical measurements including Phe-Phe dipeptide control (Supplementary Figs. 24-29 and Supplementary Table 9):

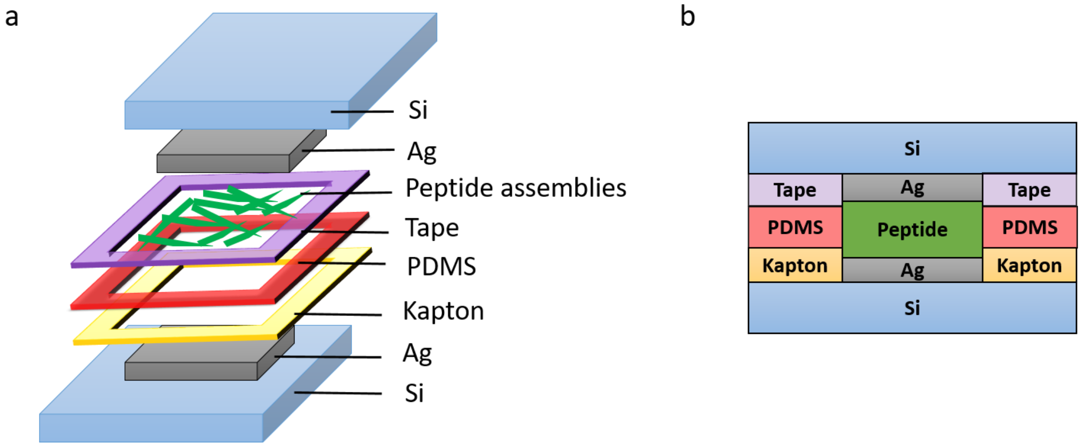

Supplementary Fig. 24| a, Schematic of the proof-of-concept power generator with peptide assemblies as the active components. **b**, Schematic cross-section diagram of the device architecture.

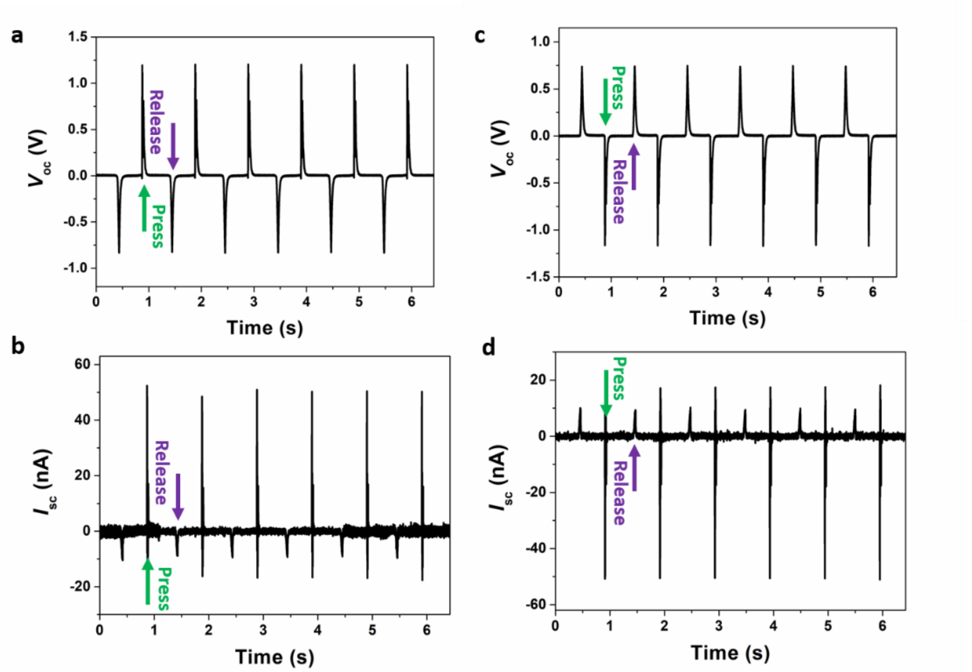

Supplementary Fig. 25| Characterization of Pro-Phe-Phe based nanogenerator. a, Open-circuit voltage and **b**, short-circuit current of piezoelectric harvester using Pro-Phe-Phe assemblies as the active layer in the forward connection upon applied force=55 N. **c**,**d**, The generated voltage output (**c**) and current output (**d**) in the reverse connection.

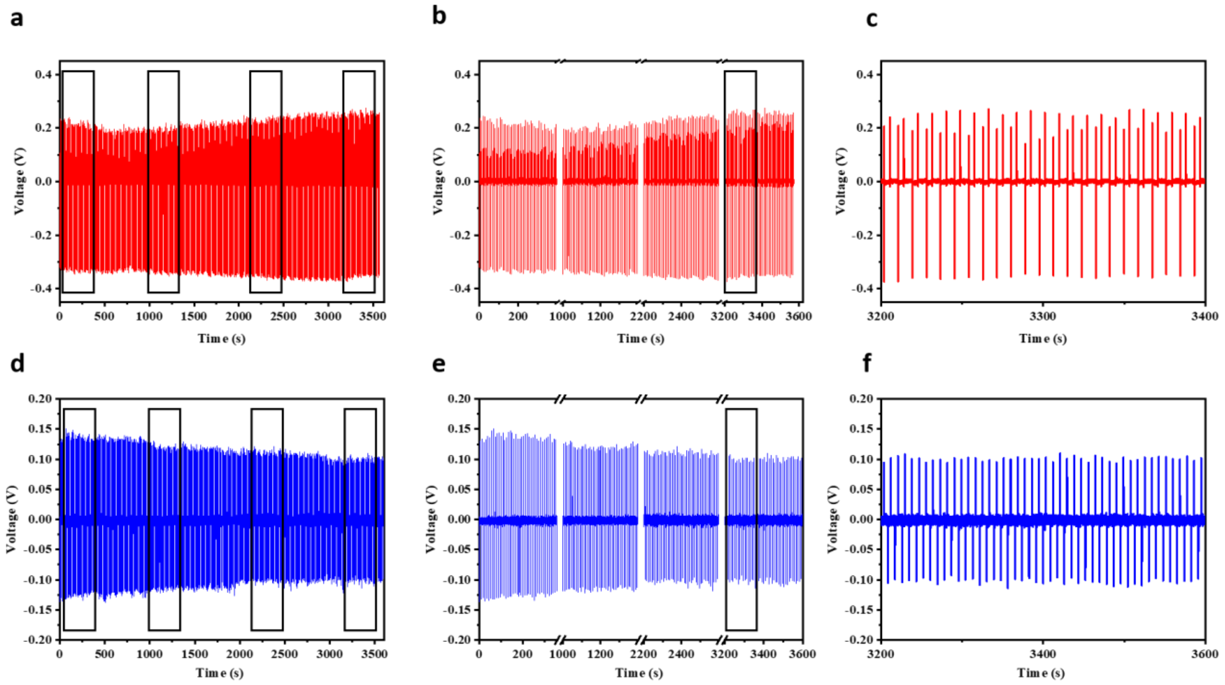

Supplementary Fig. 26| Stability testing of Hyp-Phe-Phe peptide nanogenerator. The open-circuit voltages from two different nanogenerators (a-c) and (d-f) were measured under applied force 17 N upon periodic displacement for over 60 min. The full datasets are plotted in the left hand panels with detailed views of periodic voltage response for portions (highlighted in black boxes) shown in the middle and then right panels. The peak to peak magnitude of the open-circuit voltage remained constant, demonstrating the stable performance of the device.

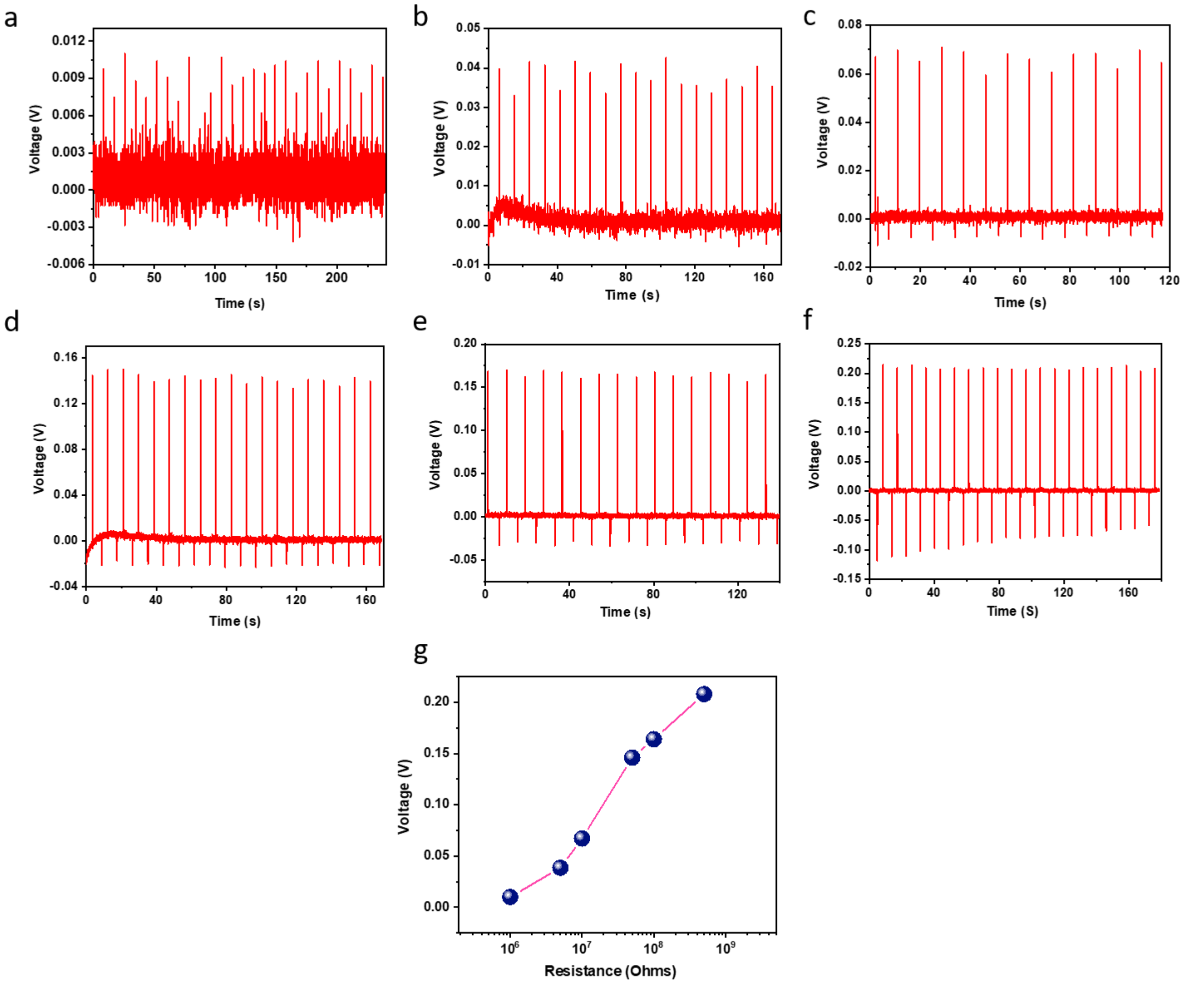

Supplementary Fig. 27| | Characterization of Hyp-Phe-Phe based nanogenerator. a-f, The open-circuit voltage measured upon applying increasing external load resistances 1 MΩ (a), 5 MΩ (b), 10 MΩ (c), 50 MΩ (d), 100 MΩ (e) and 500 MΩ (f) at the constant applied force of 17 N. g) The average voltage output as a function of external load resistance.

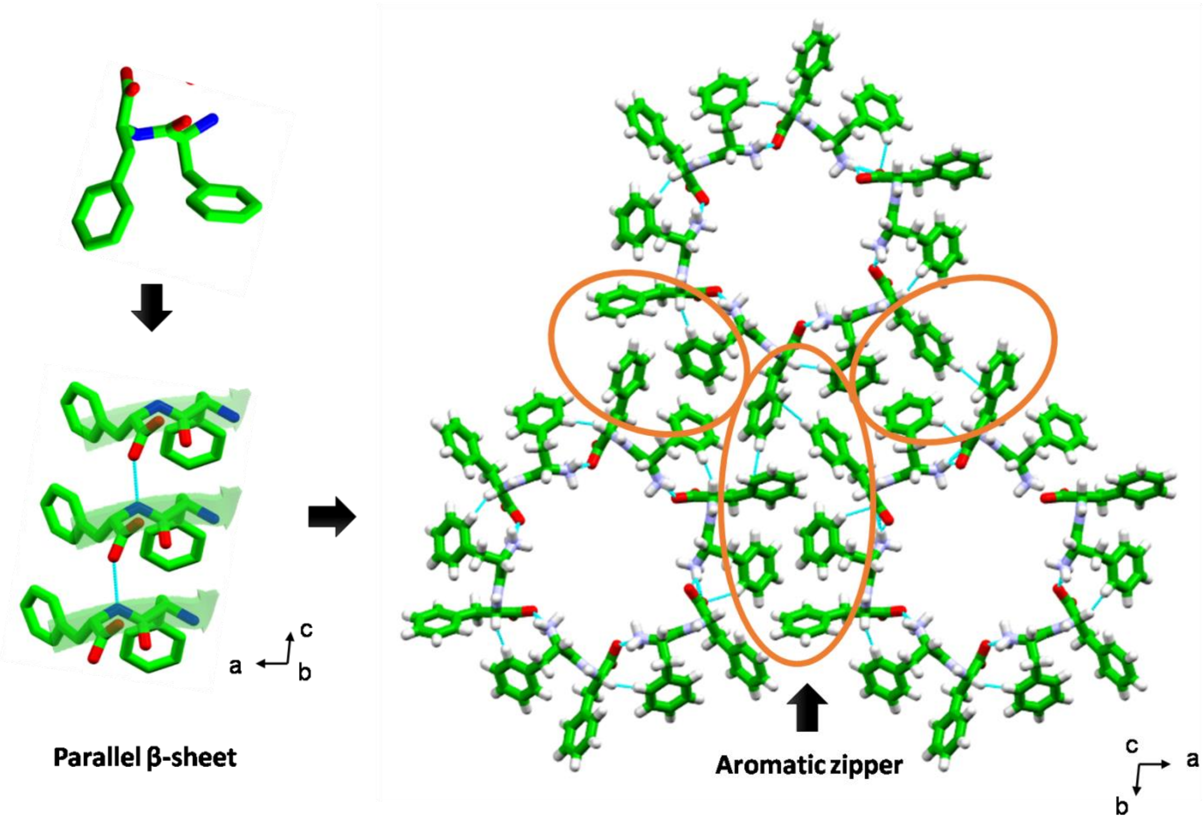

Supplementary Fig. 28| Single crystal structure of Phe-Phe, CCDC ref. no 16340[32]. Nearby molecules stack in parallel fashion to form parallel β-sheet organisation. Aromatic zipper interaction created higher order packing.

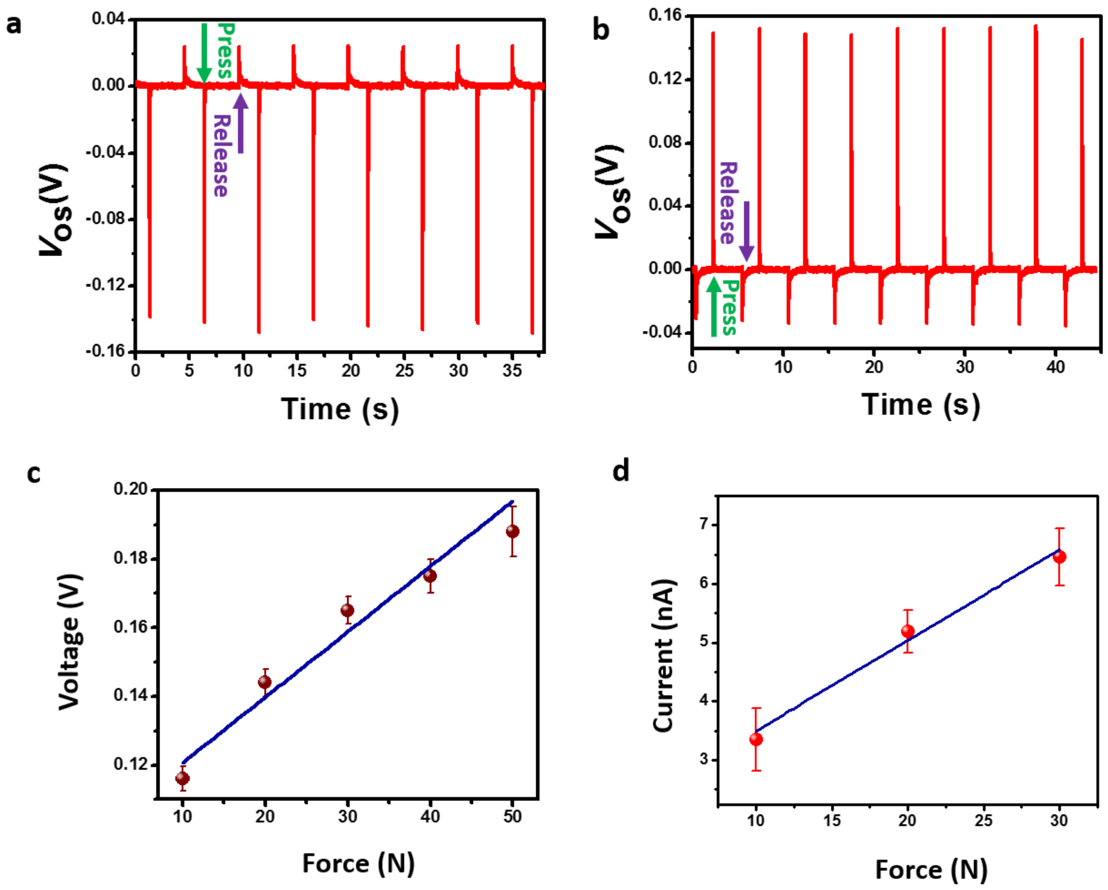

Supplementary Fig. 29| Performance of the control Phe-Phe dipeptide based nanogenerator. Open-circuit voltage of piezoelectric harvester using Phe-Phe assemblies upon applied force=23 N, in **a**, forward connection and **b**, reverse connection. **c**,**d**, Linear dependence of the voltage (**c**) and current (**d**) output on the applied force. Error bars = Standard Deviation (n=3)

Supplementary Table 9. Measured piezoelectric response of various biological, organic and inorganic nanogenerator materials.

Class of material	Material	Current (I_{sc}) and voltage (V_{oc}) output	Supplementary Reference
**Biomaterials**	Pro-Phe-Phe	52 nA/1.24 V	This work
	Hyp-Phe-Phe	39.3 nA/0.45 V	
	Phe-Phe	7.0 nA/0.2 V	
	Fish skin collagen	1.5µA-20 nA/2-4 V	8, 33
	M13 bacteriophage film	6 nA/ 0.40V	11
	Vertical phage nanopillars	9.5 nA/0.14 V	12
	Poly-L-lactic acid	Not reported / 0.9 V	14
	FF flexible	7 nA/0.6 V	34
	FF aligned	39 nA/1.4 V	16
	γ-glycine	Not reported / 0.45 V	35
**Organic materials**	Polyvinylidene-fluoride (PVDF)	3 nA/ 0.03 V	36
	Poly(vinylidenefluoride-co-trifluoroethylene)	33nA/1.3V	37
**Inorganic materials**	MoS_2	0.03 nA/ 0.015 V	38
	ZnO	0.5 nA/ 0.05 V	39
	$LiNbO_3$	9.1nA/0.46 V	40

Supplementary Section 6.

Crystal structure and DFT predicted piezoelectric response of Hyp-Leu-Phe (including Supplementary Figs. 30-37 and Supplementary Table 10-12)

Hyp-Leu-Phe.

To understand the role of aromatic moieties in high piezo response of Hyp-Phe-Phe, we modified the sequence by mutating central Phe with non-aromatic Leu. The tripeptide crystalized (Supplementary Table 10) in the $P2_1$ space group, with two tripeptide molecules in the asymmetric unit, with two side-chains arranged in the same face relative to the peptide backbone (Supplementary Fig. 30a). Single crystal structure analysis of Hyp-Leu-Phe reveled unprecedented similarity to Hyp-Phe-Phe in both backbone conformation and supramolecular organization. The allowed torsion angles of the Leu_2 residue were found to be localized within the right handed helical region of the Ramachandran plot, with φ_2 and ψ_2 values of -66.2° and -38.7° for molecule A and -72.9° and -42.4° for molecule B, respectively. In the crystallographic *b*-direction, Hyp-Leu-Phe propagated through head-to-tail intermolecular hydrogen bonds, generating a helical arrangement of the peptide backbone (Supplementary Fig. 30b).The formation of helical sheet and cross-helical architecture was also found to be similar to Hyp-Phe-Phe (Supplementary Fig. 30c). However, the only difference was found in hydrophobic dry steric zipper region which hold the interacted helical sheets to compose a cross-αlike architecture. Due to reduce number of aromatic moiety in Hyp-Leu-Phe, the density of aromatic side chain in the zipper region was found lower compare to Hyp-Phe-Phe (Supplementary Fig. 30d).

Having verified helical molecular organization, we used density functional theory (DFT) to predict the piezoelectric constants of the tripeptide (Supplementary Fig. 31 and Supplementary Table 11). Supplementary Tables 11 shows the DFT-computed piezoelectric charge (*e*), strain (*d*), and voltage (*g*) tensors of Hyp-Leu-Phe. We noticed a significant decrease in the magnitude

of the predicted piezoelectric strain constants, with $d_{max} = d_{34}$ = 3.6 pm/V, d_{25}= -3.5 pm/V,and d_{36}= -3.3 pm/V. This substantial lowered strain constant indicates the pivotal role played by aromatic residues in the electrical dipole moment and corresponding high piezoelectricity of peptide building blocks.

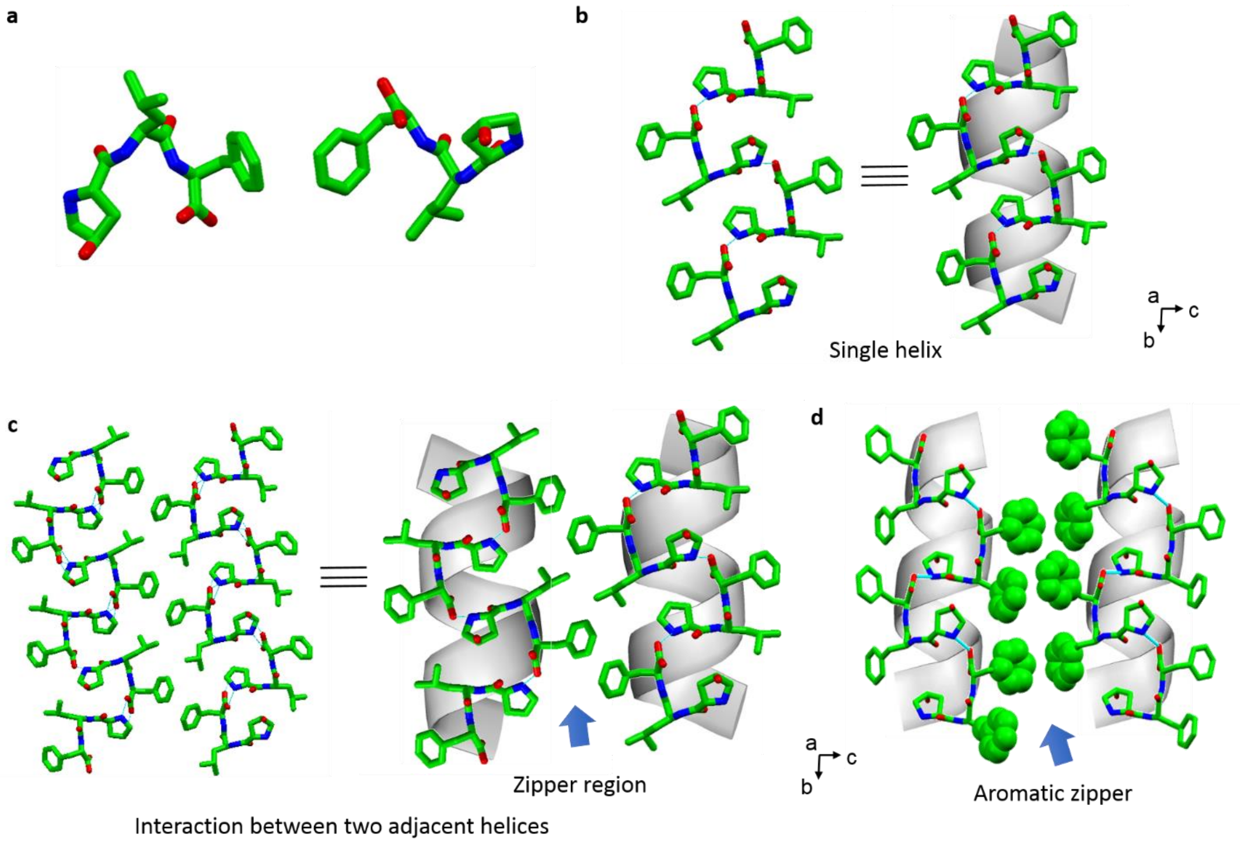

Supplementary Fig. 30| Single crystal structure of Hyp-Leu-Phe in *P2₁* space group. a, The asymmetric unit. **b**, Head-to-tail hydrogen bonds connection to produce single helical-like structural pattern viewed approximately along the *b*-axis. **c**, Interaction of nearby helices through hydrophobic zipper–like molecular packing along the *c*-direction. **d**, The aromatic density in zipper region of Hyp-Phe-Phe. For clarity, the peptide helix is superimposed over an ideal helical model in (**b**,**c**,**d**). Nitrogen and oxygen heteroatoms are designated in blue and red, respectively.

Supplementary Table 10: Data collection and refinement statistics

Experimental details:

Crystal data	Hyp-Leu-Phe
Chemical formula	C20 H29 N3 O5
Mr	391.46
Crystal system	Monoclinic
Space group	$P2_1$
a (Å)	5.4611(3)
b (Å)	11.8614(6)
c (Å)	32.0728(18)
α (°)	90
β (°)	90.781(5)
γ (°)	90
V ($Å_3$)	2077.36(19)
Z , *Z'*	4
μ (mm^{-1})	0.090
Temperature (K)	100 (2)
Data collection	
Diffractometer	XtaLAB AFC12 (RINC): Kappa dual home/near
Wavelength (Å)	0.71073
Crystal size (mm)	0.265x0.068x0.035
*T*min , *T*max	0.993,0.997
$N_{measured}$(unique)	20101(8366)
$N_{observed}$ [$I > 2\sigma(I)$]	6840
R_{int}	0.0832
θ_{max} (°)	27.102
Refinement	
R[F2 > 2σ(F2)]	0.1146
wR	0.1290
wR[*F*2 > 2σ(*F*2)]	0.2798
wR(*F*2)	0.2878
Goodness-of-fit	1.089
No. of reflections	8366
No. of parameters	506
No. of restraints	38
H-atom treatment	H-atom parameters constrained

Supplementary Table 11: Calculated piezoelectric charge tensor components e_{ij} (in units of C/m^2), strain tensor components d_{ik} (pm/V), and voltage tensor components g_{ij} (mV m/N), of Hyp-Leu-Phe.

Charge Tensor (C m^{-2})

$$\begin{pmatrix} 0 & 0 & 0 & -0.007 & 0 & 0.002 \\ 0.006 & 0.008 & -0.005 & 0 & -0.018 & 0 \\ 0 & 0 & 0 & 0.016 & 0 & -0.008 \end{pmatrix}$$

Strain Tensor (pC N^{-1})

$$\begin{pmatrix} 0 & 0 & 0 & -1.6 & 0 & 0.6 \\ 0.3 & 0.4 & -0.2 & 0 & -3.5 & 0 \\ 0 & 0 & 0 & 3.6 & 0 & -3.3 \end{pmatrix}$$

Voltage Tensor (mV m N^{-1})

$$\begin{pmatrix} 0 & 0 & 0 & -58 & 0 & 24 \\ 11 & 13 & -9 & 0 & -123 & 0 \\ 0 & 0 & 0 & 121 & 0 & -110 \end{pmatrix}$$

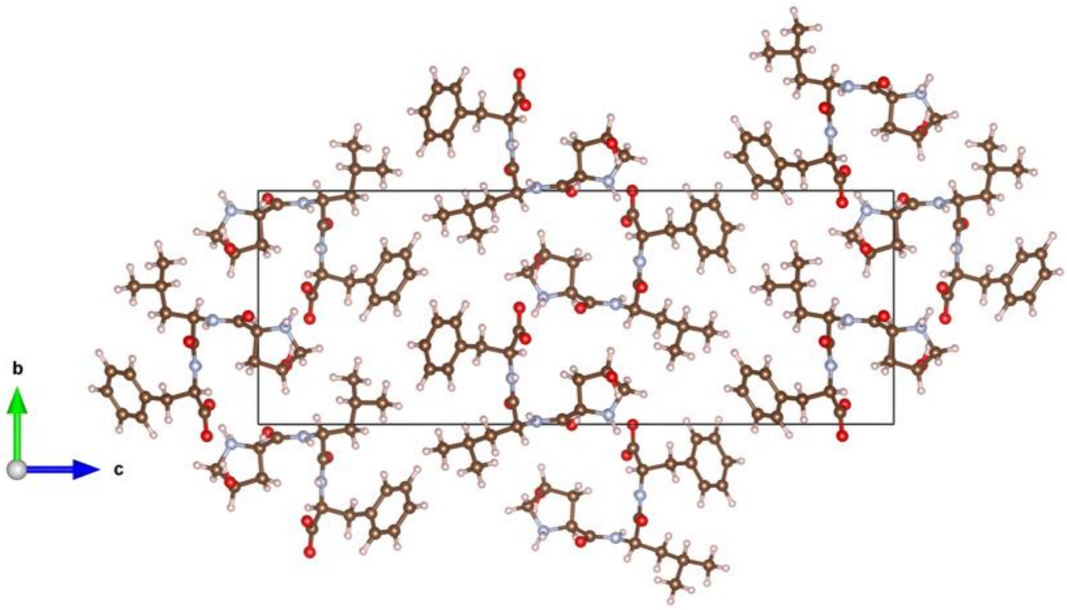

Supplementary Fig. 31| The single crystal unit cells of Hyp-Leu-Phe computed using DFT with the CP2K code (see main text Methods) are shown with the crystal dipole moment overlaid as a dark green arrow. Tripeptide is represented by ball and sticks.

Supplementary Table 12: List of peptides studied and their components, molecular organization and piezoelectricity relation.

Peptide	Conformation	Piezoelectric constant **(pm/V)**
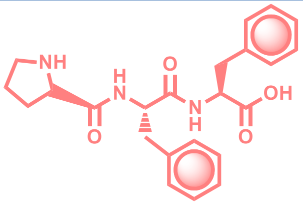 **Pro-Phe-Phe**	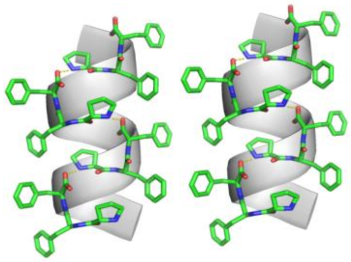 **Helix**	**1.9**
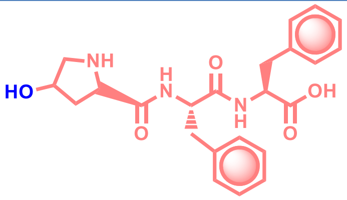 **Hyp-Phe-Phe**	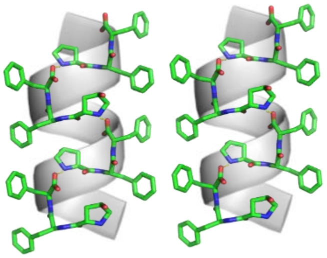 **Helix**	**27.3**
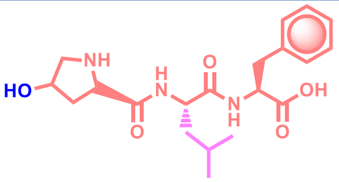 **Hyp-Leu-Phe**	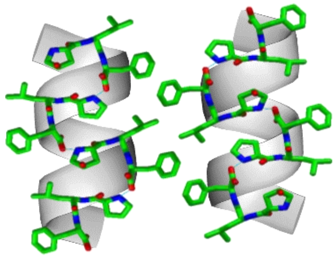 **Helix**	**3.6**
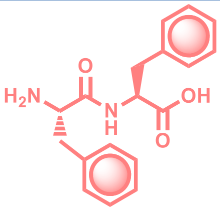 **Phe-Phe**	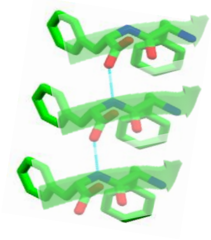 **β-sheet**	**9.9**

a

MASS SPECTROMETRY REPORT

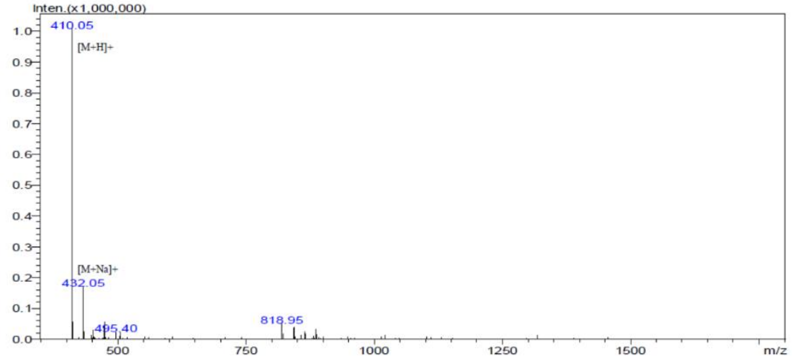

Sample Description		Instrument	SHIMADZU LCMS-2010EV		
Analyzed date:	2017-11-27	Probe:	ESI	Probe Bias:	+4.5kv
Analyst:	Wu	Nebulizer Gas Flow:	1.5L/min	Detector:	1.5kv
Sample:	T-1795	CDL:	-20.0v	T. Flow:	0.2ml/min
M.W.:	409.5	CDL Temp.:	250 °C	B. Conc.:	50%H2O/50%ACN
Sequence	PFF	Block Temp.:	200 °C		

b

HPLC REPORT

Sample: T-1795 Analyzed date: 2017-11-27
Sequence PFF
Lot. No.: DG-64963
Column: 4.6*250mm, Welch CX-C18 5um
Solvent A A: 0.1% Trifluoroacetic Acid in 100% Acetonitrile
Solvent B B: 0.1% Trifluoroacetic Acid in 100% Water
Gradient:

	A	B
0.0min	25%	75%
25.0min	50%	50%
25.1min	100%	0%
30.0min	Stop	

Volume: 20μl
Wavelength: 220nm
Flow rate: 1.0ml/min

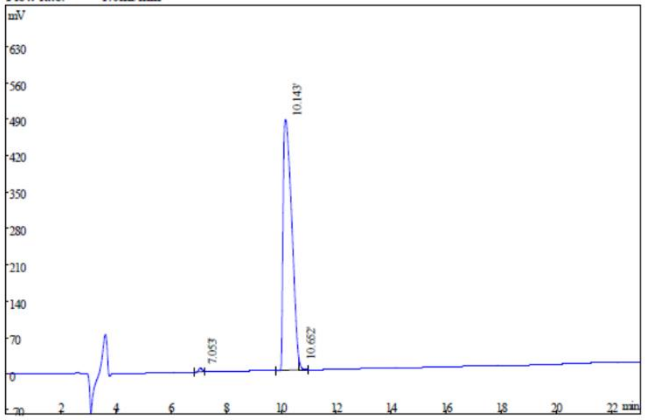

Rank	Time	Conc.	Area
1	7.053	0.5649	57112
2	10.143	98.98	10006303
3	10.652	0.4551	46009
Total		100	10109424

Supplementary Fig. 32| Characterization of Pro-Phe-Phe. a, Mass Spectra, **b,** HPLC trace.

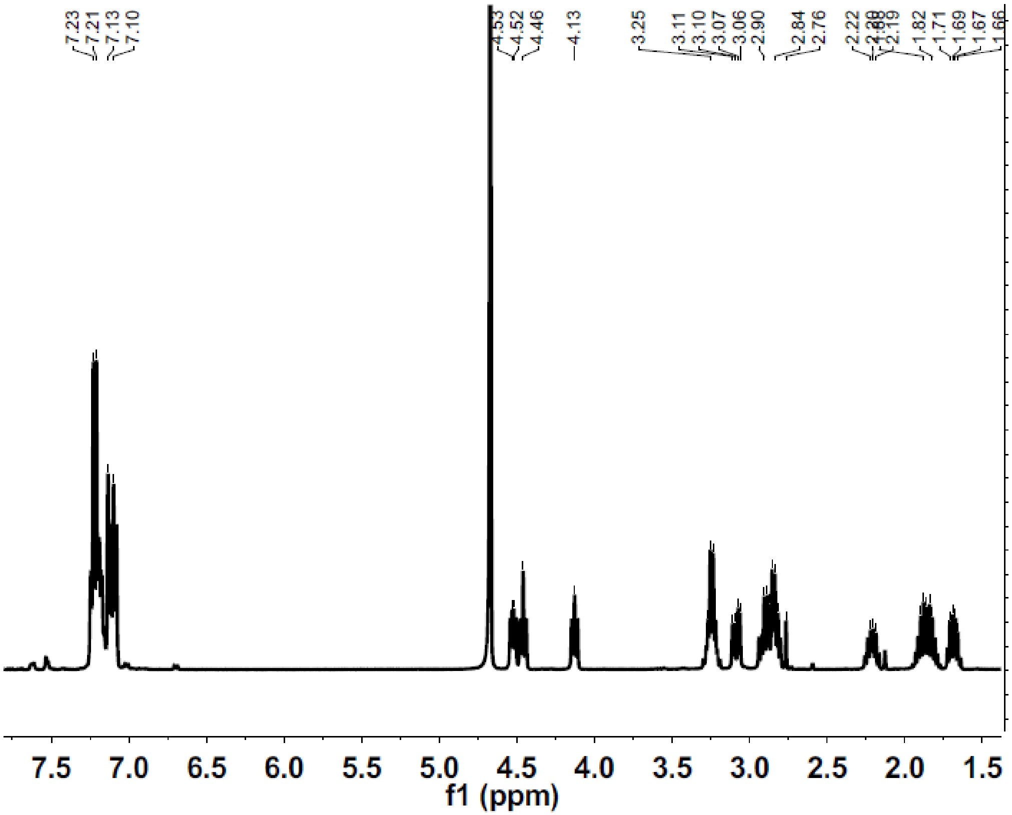

Supplementary Fig. 33| Characterization of Pro-Phe-Phe. ^{1}H NMR spectra.

a

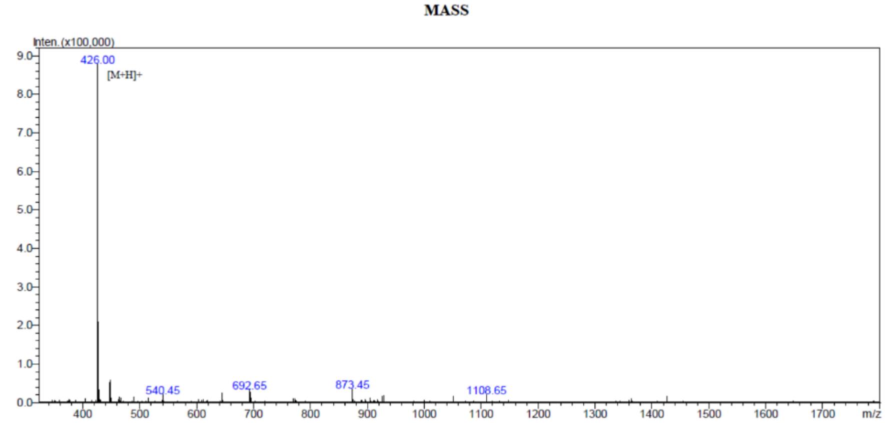

b

HPLC

Sample:	T-3137	Analyzed date:	2019-03-27
Sequence:	Hyp-Phe-Phe		
Lot. No.:	DG-72769		
Column:	COSMOSIL, 4.6*250mm, 5μm		
Solvent A	A: 0.1% Trifluoroacetic Acid in 100% Acetonitrile		
Solvent B	B: 0.1% Trifluoroacetic Acid in 100% Water		

Gradient:

	A	B
0.0min	24%	76%
25.0min	49%	51%
25.1min	100%	0%
30.0min	Stop	

Volume: 20μl
Wavelength: 220nm
Flow rate: 1.0ml/min

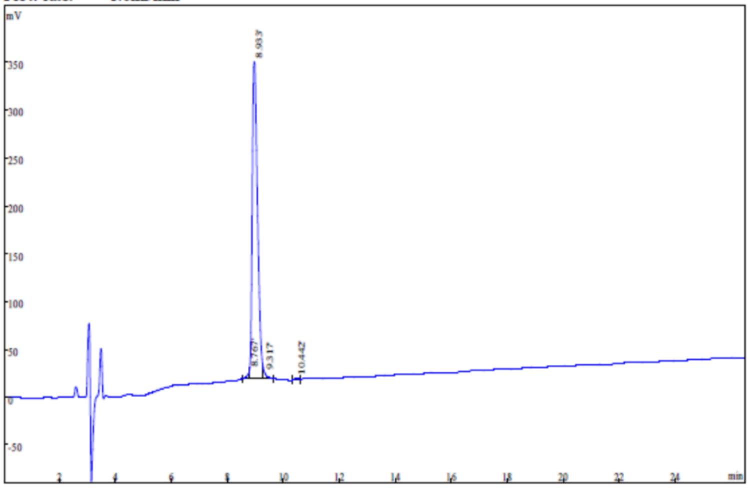

Rank	Time	Area	Conc.
1	8.767	36820	0.8989
2	8.933	3989761	97.41
3	9.317	60509	1.467
4	10.442	9212	0.2249
Total			100

Supplementary Fig. 34| Characterization of Hyp-Phe-Phe. a, Mass Spectra, **b,** HPLC trace.

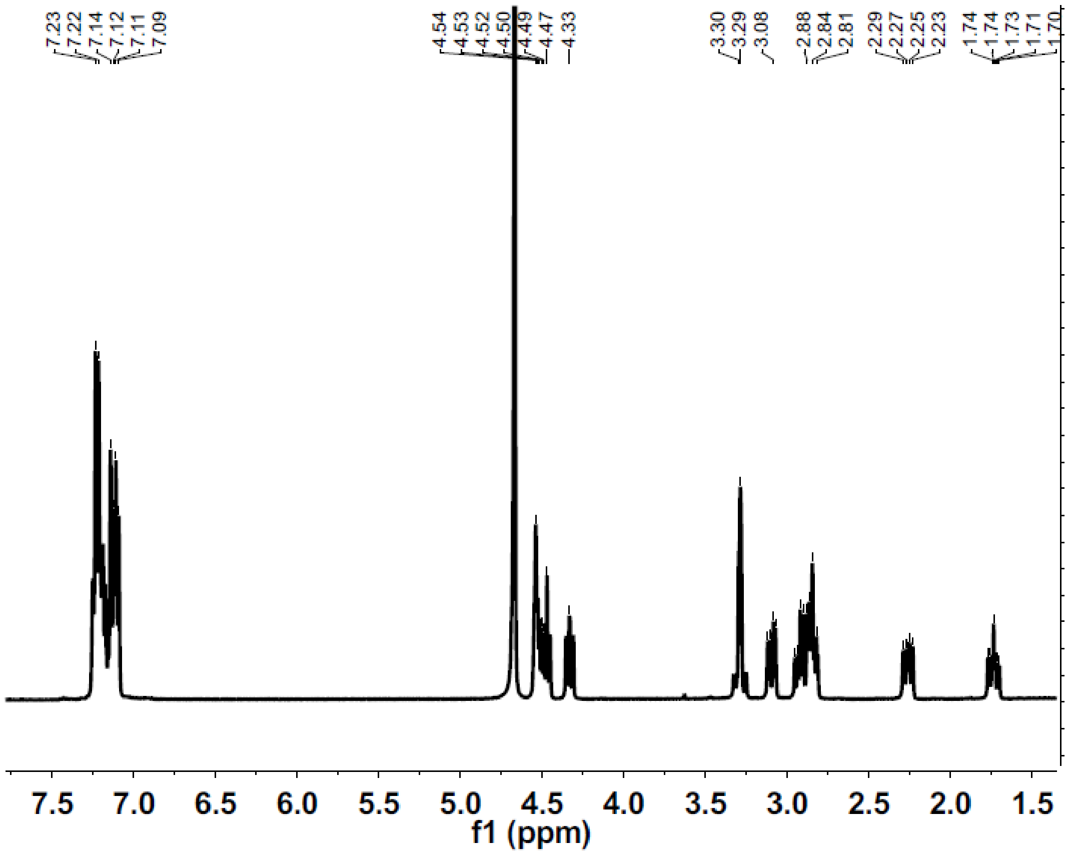

Supplementary Fig. 35| Characterization of Hyp-Phe-Phe. ^{1}H NMR spectra.

a

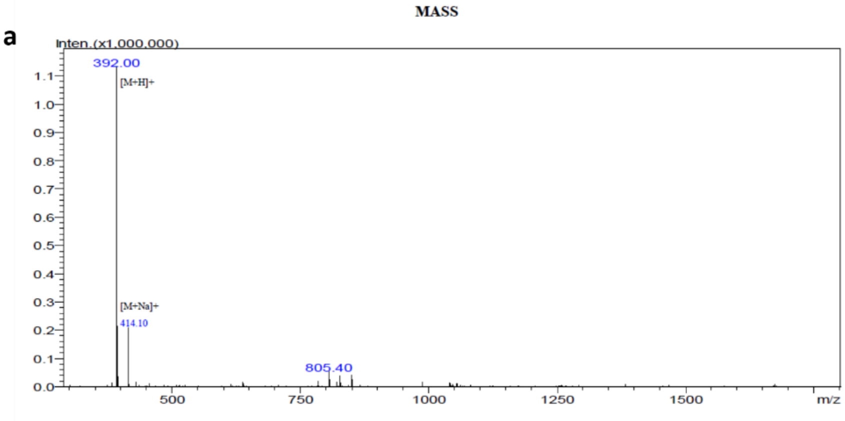

b

HPLC

Sample:	T-3190	Analyzed date:	2019-06-04
Sequence:	Hyp-Leu-Phe		
Lot. No.:	DG-73016		
Column:	Kromasil C18, 4.6*250mm, 5μm		
Solvent A	A: 0.1% Trifluoroacetic Acid in 100% Acetonitrile		
Solvent B	B: 0.1% Trifluoroacetic Acid in 100% Water		

Gradient:

	A	B
0.0min	20%	80%
25.0min	45%	55%
25.1min	100%	0%
30.0min	Stop	

Volume: 20μl

Wavelength: 220nm

Flow rate: 1.0ml/min

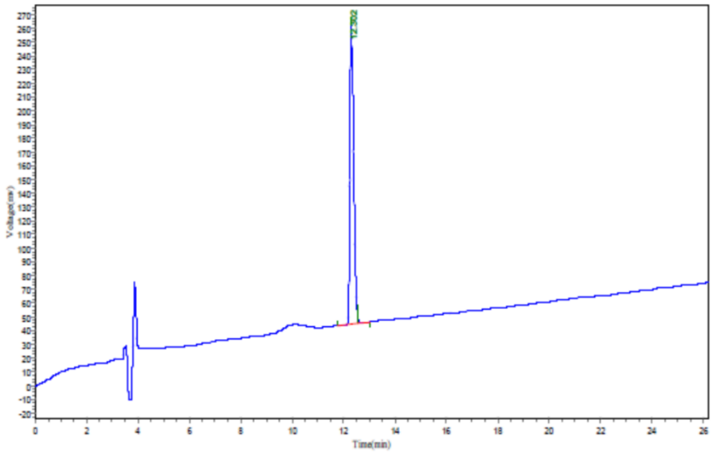

Rank	Time	Area	Conc.
1	12.302	2152508.500	98.6950
2	12.302	28461.805	1.3050
Total		2180970.305	100

Supplementary Fig. 36| Characterization of Hyp-Leu-Phe. a, Mass Spectra, **b,** HPLC trace.

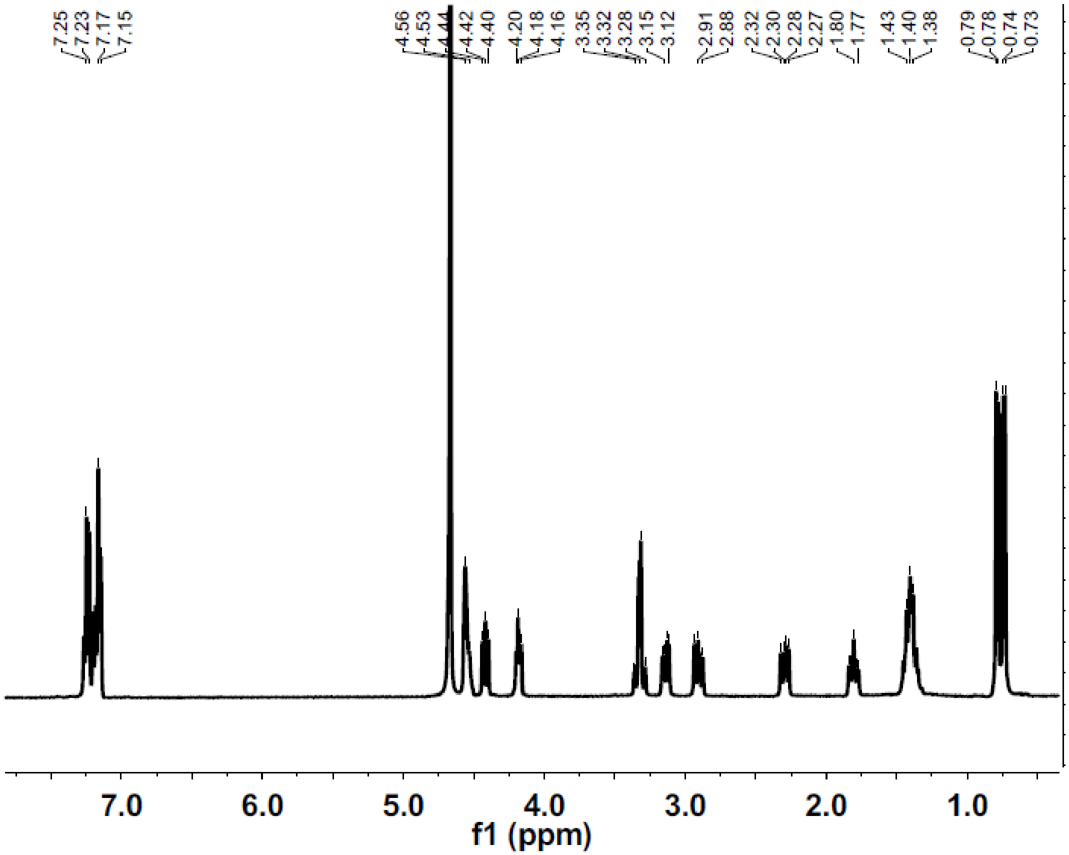

Supplementary Fig. 37| Characterization of Hyp-Leu-Phe. ^{1}H NMR spectra.

Supplementary References

1. Lübbe, J. *et al*. Determining cantilever stiffness from thermal noise. *Beilstein J. Nanotechnol* **4**, 227-233 (2013).
2. Chung, P. C. *et al*. The elastic mechanical response of supported thin polymer films. *Langmuir* **30**, 15200-15205 (2014).
3. Maugid, D., Contact, adhesion and rupture of elastic solids. Springer-Verlag: Berlin, 2000.
4. Usov, I. & Mezzenga, R. FibreApp: an open-source Software for tracking and analyzing polymers, filaments, biomacromolecules, and fibrous objects. *Macromolecules* ***48***, 1269-1280 (2015).
5. Gundjian, A. A. & Chen, H. L. Standardization and interpretation of the electromechanical properties of bone. *IEEE Trans. Biomed. Eng. BME* **21**, 177–182(1974).
6. Zhou, Z., Qian, D. &Minary-Jolandan, M. Molecular mechanism of polarization and piezoelectric effect in super-twisted collagen, *ACS Biomater. Sci. Eng*. **2**, 929–936 (2016).
7. Minary-Jolandan,M.& Yu,M.-F. Nanoscale characterization of isolated individual type I collagen fibrils: polarization and piezoelectricity. *Nanotechnology* **20**, 085706–085712(2009).
8. Ghosh, S. K. & Mandal, D. High-performance bio-piezoelectric nanogenerator made with fish scale, *Appl. Phys. Lett.***109**, 103701 (2016).
9. Denning, D. et al. Piezoelectric tensor of collagen fibrils determined at the nanoscale, *ACS Biomater. Sci. Eng*. **3**, 929–935 (2017).

10. Guerin, S., Tofail, A. M.S. & Thompson, D. Deconstructing collagen piezoelectricity using alanine-hydroxyproline-glycine building blocks. *Nanoscale*, **10**, 9653-9663 (2018).
11. Lee, B. Y. *et al.* Virus-based piezoelectric energy generation. *Nat. Nanotech*. **7**, 351-356 (2012).
12. Shin, D.-M. et al. Bioinspired piezoelectric nanogenerators based on vertically aligned phage nanopillars. *Energy Environ. Sci.* **8**, 3198-3203 (2015).
13. Farrar, D. et al. Permanent polarity and piezoelectricity of electrospun α -helical poly(α -amino acid) fibers**,** *Adv. Mater.* **23**, 3954–3958 (2011)**.**
14. Curry, E. J. Biodegradable piezoelectric force sensor, *Proc. Natl. Acad. Sci. U.S.A.***115**, 909–914 (2018).
15. Nguyen, V., Jenkins, K. & Yang, R. Epitaxial growth of vertically aligned piezoelectric diphenylalanine peptide microrods with uniform polarization. *Nano Energy* **17**, 323–329 (2015).
16. Nguyen, V., Zhu, R., Jenkins, K.& Yang, R. Self-assembly of diphenylalanine peptide with controlled polarization for power generation. *Nat. Commun.**7***, 13566–13572 (2016).
17. Joseph, J., Singh, S. G. & Vanjari, S. R. K. Piezoelectric Micromachined Ultrasonic Transducer Using Silk Piezoelectric Thin Film. *IEEE Electron Device Letters* **39**, 749-752 (2018).
18. Ueberschlag, P. PVDF piezoelectric polymer. Sensor review (2001).
19. H. Joffe, D. Berlincourt, H. Krueger and L. Shiozawa. Piezoelectric properties of cadmium sulfide crystals. 14th Annual Symposium on Frequency Control, Atlantic City, NJ, USA, 1960, pp. 19-23, doi: 10.1109/FREQ.1960.199429.
20. Denishev, K. Some metal oxides and their applications for creation of Microsystems

(MEMS) and Energy Harvesting Devices (EHD). *J. Phys. Conf. Ser*. **764**, 012003 (2016).

21. Jungk, T., Hoffmann, A. & Soergel, E. Contrast mechanisms for the detection of ferroelectric domains with scanning force microscopy. *New J. Phys*. **11**, 033029 (2009).
22. Lueng, C.M., Chan, H. L. W., Surya,C., Choy,C. L. Piezoelectric coefficient of aluminum nitride and gallium nitride. *J. Appl. Phys.* **88**, 5360–5363 (2000).
23. Wang, Yaojin, *et al*. Large piezoelectricity in ternary lead-free single crystals. *Adv. Electron. Mater*. 6.1 (2020): 1900949.
24. Tateyama, A. *et al*. Good piezoelectricity of self-polarized thick epitaxial (K, Na) NbO3 films grown below the Curie temperature (240° C) using a hydrothermal method. *Appl. Phys. Lett*. **117**, 142903 (2020).
25. Zhao, Q. *et al*. Flexible textured MnO_2 nanorods/PVDF hybrid films with superior piezoelectric performance for energy harvesting application. *Compos. Sci. Technol*. **199**, 108330 (2020).
26. Chelu, M. *et al*. High-quality PMMA/ZnO NWs piezoelectric coating on rigid and flexible metallic substrates. *Appl. Surf. Sci*. **529**, 147135 (2020).
27. Scrymgeour, D. A. & Hsu, J. W. Correlated piezoelectric and electrical properties in individual ZnO nanorods. *Nano letters* **8**, 2204-2209 (2008).
28. Luo, Y. *et al.* Nanoshell tubes of ferroelectric lead zirconate titanate and barium titanate. *Appl. Phys. Lett*. **83**, 440-442 (2003).
29. Minary-Jolandan, M., Bernal, R. A., Kuljanishvili, I., Parpoil, V. & Espinosa, H. D. Individual GaN nanowires exhibit strong piezoelectricity in 3D. *Nano letters* **12**, 970-976 (2012).
30. Fei, R., Li, W., Li, J. & Yang, L. Giant piezoelectricity of monolayer group IV

monochalcogenides: SnSe, SnS, GeSe, and GeS. *Appl. Phys. Lett* **107**, 173104 (2015).

31. Fei, R., Li, W., Li, J. & Yang, L. Giant piezoelectricity of monolayer group IV monochalcogenides: SnSe, SnS, GeSe, and GeS. *Appl. Phys. Lett* **107**, 173104 (2015).
32. Görbitz, C. H. Nanotube formation by hydrophobic dipeptides, *Chem. Eur. J*. **7**, 5153 – 5159 (2001).
33. Ghosh, S. K. & Mandal, D. Sustainable energy generation from piezoelectric biomaterial fornoninvasive physiological signal monitoring, *ACS Sustainable Chem. Eng*. **5**, 8836−8843 (2017).
34. Jenkins, K., Kelly, S., Nguyen, V., Wu, Y. & Yang, R. Piezoelectric diphenylalanine peptide for greatly improved flexible nanogenerators, *Nano Energy* **51**, 317–323 (2018).
35. Guerin, S. et al. Control of piezoelectricity in amino acids by supramolecular packing. *Nat. Mater.***17**, 180–186 (2018).
36. Chang, C., Tran, V. H., Wang, J., Fuh, Y.-K. & Lin, L. Direct-write piezoelectric polymericnanogenerator with high energy conversion efficiency, *Nano Lett*. 10, 726-731 (2010).
37. Persano, L. et al. High performance piezoelectric devices based on aligned arrays of nanofibers of poly(vinylidenefluoride-co-trifluoroethylene), *Nat. Commun.* 4:1633 (|2012).
38. Wu, W. et al. Piezoelectricity of singleatomic-layer MoS_2 for energy conversion and piezotronics. *Nature* **514**, 470–474 (2014).
39. Li, Z.,Zhu, G., Yang, R., Wang, A. C. & Wang, Z. L. Muscle-driven in vivo nanogenerator. *Adv. Mater.* **22**, 2534-2537 (2010).
40. Yun, B. K. et al. Lead-free $LiNbO_3$ nanowire-based nanocomposite for piezoelectric power generation. *Nanoscale Res. Lett*. **9**, 4 (2014).
